# Microglial depletion alters the brain neuroimmune response to acute binge ethanol withdrawal

**DOI:** 10.1186/s12974-017-0856-z

**Published:** 2017-04-20

**Authors:** T. Jordan Walter, Fulton T. Crews

**Affiliations:** 1 Department of Pharmacology, University of North Carolina at Chapel Hill, Chapel Hill, NC 27599 USA; 2 Bowles Center for Alcohol Studies, University of North Carolina at Chapel Hill, Thurston-Bowles Building, Chapel Hill, NC 27599 USA

**Keywords:** Microglia, Binge, Ethanol, Withdrawal, Neuroimmune, Pro-inflammatory, Anti-inflammatory

## Abstract

**Background:**

Recent studies have implicated microglia—the resident immune cells of the brain—in the pathophysiology of alcoholism. Indeed, post-mortem alcoholic brains show increased microglial markers and increased immune gene expression; however, the effects of ethanol on microglial functioning and how this impacts the brain remain unclear. In this present study, we investigate the effects of acute binge ethanol on microglia and how microglial depletion changes the brain neuroimmune response to acute binge ethanol withdrawal.

**Methods:**

C57BL/6J mice were treated intragastrically with acute binge ethanol for time course and dose-response studies. Cultured mouse BV2 microglia-like cells were treated with ethanol in vitro for time course studies. Mice were also administered the colony stimulating factor 1 receptor (CSF1R) inhibitor PLX5622 to deplete microglia from the brain. These mice were subsequently treated with acute binge ethanol and sacrificed during withdrawal. Brain and BV2 mRNA were isolated and assessed using RT-PCR to examine expression of microglial and neuroimmune genes.

**Results:**

Acute binge ethanol biphasically changed microglial (e.g., Iba1, CD68) gene expression, with initial decreases during intoxication and subsequent increases during withdrawal. Acute ethanol withdrawal dose dependently increased neuroimmune gene (e.g., TNFα, Ccl2, IL-1ra, IL-4) expression beginning at high doses. BV2 cells showed biphasic changes in pro-inflammatory (e.g., TNFα, Ccl2) gene expression following ethanol treatment in vitro. Administration of PLX5622 depleted microglia from the brains of mice. Although some neuroimmune genes were reduced by microglial depletion, many others were unchanged. Microglial depletion blunted pro-inflammatory (e.g., TNFα, Ccl2) gene expression and enhanced anti-inflammatory (e.g., IL-1ra, IL-4) gene expression during acute binge ethanol withdrawal.

**Conclusions:**

These studies find acute binge ethanol withdrawal increases microglial and neuroimmune gene expression. Ethanol exposure also increases microglial pro-inflammatory gene expression in vitro. Furthermore, microglial depletion decreases expression of microglia-specific genes but has little effect on expression of many other neuroimmune signaling genes. Microglial depletion blunted the acute binge ethanol withdrawal induction of pro-inflammatory genes and enhanced induction of anti-inflammatory genes. These findings indicate microglia impact the brain response to acute binge ethanol withdrawal.

**Electronic supplementary material:**

The online version of this article (doi:10.1186/s12974-017-0856-z) contains supplementary material, which is available to authorized users.

## Background

Alcohol use disorders (AUDs) are a common mental health disease in which individuals drink excessive amounts of alcohol despite negative consequences. Many individuals with AUDs engage in heavy patterns of alcohol consumption known as binge drinking, defined as drinking to a blood alcohol concentration (BAC) greater than 80 mg/dL [1]. However, many studies in humans have documented even more extreme binge drinking leading to BACs around 400 mg/dL [2–4]. Excessive alcohol consumption has several detrimental effects on the brain, contributing to neurodegeneration [5], neuronal dysfunction [6], and various other pathologies. Research even suggests excessive alcohol changes the brain to promote further alcohol consumption [7]. However, much remains unknown regarding the mechanisms by which excessive alcohol use negatively impacts the brain.

Interestingly, recent studies have implicated the innate immune system in the pathophysiology of alcohol use disorders. Deletion of innate immune genes such as *IL1rn* or *IL6* decreases voluntary alcohol consumption [8, 9]. Furthermore, alcohol treatment increases innate immune gene expression in the brain [10, 11]. Even a single, heavy dose of alcohol increases expression of innate immune genes such as TNFα and Ccl2 in the brain [12]. Microglia are the resident innate immune cells of the brain and thought to be the primary mediators of the brain immune response to alcohol. Indeed, microglial markers such as Iba1 are increased in the brains of post-mortem alcoholics [13, 14], consistent with microglial activation. However, there are multiple types of microglial activation [15], with functional effects ranging from pro-inflammatory and destructive to anti-inflammatory and healing, and increased markers such as Iba1 do not indicate activation type [16, 17]. While a few studies have begun to investigate the effects of alcohol on microglial function in vivo [18], much remains unknown. In vitro studies in human monocytes suggest ethanol may have complex, dynamic effects, as short-term ethanol exposure decreases inflammatory signaling, while long-term ethanol exposure increases inflammatory signaling [19]. Even the microglial response to a single episode of ethanol exposure and withdrawal in vivo is not fully understood. Therefore, in this study, we sought to investigate the dynamic effects of acute binge ethanol on microglia in vivo.

Understanding the effects of ethanol on microglia is critical, as microglia interact extensively with other cell types of the brain to impact brain function. Microglia can alter synapse formation through brain-derived neurotrophic factor (BDNF) [20], synapse elimination through complement-dependent phagocytosis [21], and synaptic plasticity through release of cytokines such as TNFα [22]. Furthermore, microglia can either promote or inhibit neurogenesis depending on their activation state [23, 24]. Activated microglia can also protect against neuronal cell death [25] or contribute to neuronal cell death [26, 27] depending on whether they are activated to a trophic or inflammatory activation state. Indeed, ethanol-induced changes in microglia may have an important impact on brain functioning and ultimately behavior. Interestingly, recent research has identified a new pharmacological tool for studying the role of microglia in vivo. Inhibitors of the colony stimulating factor 1 receptor (CSF1R) deplete microglia from the brain without known detrimental effects or behavioral changes [28]. This allows for an opportunity to examine the function of microglia in vivo in a novel way. We sought to utilize this method to investigate the effects of microglial depletion on both the normal brain and the ethanol-treated brain. Furthermore, microglial depletion allows for examination of the contributions of non-microglial cells to the brain response to ethanol. We examined the effect of acute binge ethanol and microglial depletion on brain expression of microglial genes, pro-inflammatory genes, anti-inflammatory genes, and various other genes including Toll-like receptors and death receptors. We hypothesized that acute binge ethanol would cause time and dose-dependent changes in microglial and neuroimmune gene expression and that microglial depletion would alter brain neuroimmune gene expression. We further hypothesized that microglial depletion would alter the brain neuroimmune response to acute binge ethanol withdrawal.

## Methods

### Animals

Male C57BL/6J mice were ordered from the Jackson Laboratory and housed in an animal facility at the University of North Carolina at Chapel Hill. All mice were group housed (*n* = 3–4) in a temperature- (20 °C) and humidity-controlled vivarium on a 12-h/12-h light/dark cycle (light onset at 0700 h) and provided ad libitum access to food and water. Experimental procedures were approved by the Institutional Animal Care and Use Committee of the University of North Carolina at Chapel Hill and conducted in accordance with National Institutes of Health regulations for the care and use of animals. For an overview of all animal experiments, see Additional file 1: Figure S1. 

### Mouse time course and dose-response experiments

For the time course experiment, 12-week-old mice were gavaged with either ethanol (6 g/kg, 25% *v*/*v*) or a comparable volume of water and sacrificed 6, 12, 18, 24, or 48 h later. A group of non-gavaged “0 h” mice was included as a control. All mice were sacrificed mid-morning (9–10 AM). A parallel group of mice was gavaged with ethanol (6 g/kg, 25% *v*/*v*), and tail blood was collected at 1, 6, 12, and 18 h for the determination of blood alcohol concentrations (BACs). For the dose-response experiment, 12-week-old mice were gavaged with ethanol (3, 4.5, or 6 g/kg, 25% *v*/*v*) or a comparable volume of water and sacrificed 18 h later. Tail blood was collected at 1 h for the determination of BACs. All mice were sacrificed mid-morning (9–10 AM). BACs were determined using a GL5 Analyzer (Analox; London, UK).

### Cell culture time course experiment

The BV2-immortalized mouse microglial cell line was maintained in DMEM supplemented with 10% FBS, 2 mM of L-alanyl-L-glutamine dipeptide (GlutaMAX, ThermoFisher, 35050061), 100 units/mL of penicillin, 100 μg/mL of streptomycin, and 0.25 μg/mL of amphotericin B (Antibiotic-Antimycotic 100×, ThermoFisher, 15240062). Cells were maintained in a humidified incubator with 5% CO_2_. For time course experiments, BV2 cells were plated at a density of 1.5 × 10^5^ cells/well in 6-well plates. After plating, cells were allowed a few hours to adhere and media was replaced with DMEM supplemented with 2% FBS, 2 mM of L-alanyl-L-glutamine dipeptide, 100 units/mL of penicillin, 100 μg/mL of streptomycin, and 0.25 μg/mL of amphotericin B. The cells were then allowed to incubate overnight. The next day, cells were treated with PBS or 85 mM ethanol in PBS at noon (24-hr time point), 6 PM (18-hr time point), midnight (12-hr time point), 6 AM the next day (6-hr time point), or 10:30 AM the next day (1.5-hr time point). At noon the next day, media were collected for analysis of ethanol concentrations and TRI reagent was added to the cells. Total mRNA was extracted as described below. For evaporation experiments, cells were placed in the incubator without vaporized ethanol in the chamber, thereby allowing media ethanol to evaporate over time. For continuous ethanol exposure experiments, cells were placed in the incubator with vaporized ethanol to keep media ethanol concentrations constant. Ethanol was vaporized into the incubator by placing a beaker with 200 mL of 4% ethanol into the chamber.

### Microglial depletion experiment

The CSF1R inhibitor PLX5622 was provided by Plexxikon Inc. (Berkeley, CA) formulated at a dose of 1200 mg/kg in AIN-76A chow by Research Diets (New Brunswick, NJ). Control chow was also provided. Twelve-week-old mice received either PLX5622 chow or control chow for 3 weeks. Mice (average weight 27 g; range 23–30 g) were then acutely gavaged with ethanol (6 g/kg, 25% *v*/*v*, i.g.) or an equivalent volume of water and sacrificed 18 h later (at 15 weeks of age). All mice were sacrificed mid-morning (9–10 AM).

### mRNA isolation, reverse transcription, and RT-PCR

Total mRNA was extracted from frozen half-brains by homogenization in TRI reagent (Sigma-Aldrich, St. Louis, MO, Cat. # T9424) following the single-step method [29]. Total mRNA was reverse transcribed. Primers used for RT-PCR are listed in Table 1. In all experiments, 18S rRNA was used as a reference gene. SYBR Green PCR Master Mix (Life Technologies, Carlsbad, CA, Cat. # 4367659) was used for the RT-PCR. The real-time PCR was run with an initial activation for 10 min at 95 °C, followed by 40 cycles of denaturation (95 °C, 15 s), annealing/extension (57–58 °C, 1 min), and finally a melt curve. The threshold cycle (*C*_T_) of each target product was determined and the ΔΔ*C*_T_ method was used to calculate the percent change compared to the control group.

**Table 1:** List of primer sequences used in this study

18S F:	5'-GGTAACCCGTTGAACCCCAT
18S R:	5'-CAACGCAAGCTTATGACCCG
Iba1 F:	5'-GGATTTGCAGGGAGGAAAAG
Iba1 R:	5'-TGGGATCATCGAGGAATTG
CD68 F:	5'-TGTCTGATCTTGCTAGGACCG
CD68 R:	5'-GAGAGTAACGGCCTTTTTGTGA
TNFα F:	5'-GACCCTCACACTCAGATCATCTTCT
TNFα R:	5'-CCTCCACTTGGTGGTTTGCT
Ccl2 F:	5'-CCAGCCTACTCATTGGGAT
Ccl2 R:	5'-GGGCCTGCTGTTCACAGTT
IL-1ra F:	5'- CTGTTGGCTGGCCTAATCCC
IL-1ra R:	5'-GCTTTGAACAAGCACCTGCC
IL-4 F:	5'-TGGGTCTCAACCCCCAGCTAGT
IL-4 R:	5'-TGCATGGCGTCCCTTCTCCTGT
IL-10 F:	5'-GCTCTTACTGACTGGCATGAG
IL-10 R:	5'-CGCAGCTCTAGGAGCATGTG
Arg1 F:	5'-TTAGGCCAAGGTGCTTGCTGCC
Arg1 R:	5'-TACCATGGCCCTGAGGAGGTTC
CD11b F:	5'-GAGGCCCCCAGGACTTTAAC
CD11b R:	5'-CTTCTTGGTGAGCGGGTTCT
CX_3_CR1 F:	5'-TCTTCACGTTCGGTCTGGTG
CX_3_CR1 R:	5'- TGCACTGTCCGGTTGTTCAT
CSF1R F:	5'-GTCCACGGCTCATGCTGAT
CSF1R R:	5'-GTGAGTACAGGCTCCCAAGAG
CD86 F:	5'-ACGATGGACCCCAGATGCACCA
CD86 R:	5'-GCGTCTCCACGGAAACAGCA
CD206 F:	5'-TCAGCTATTGGACGCGAGGCA
CD206 R:	5'-TCCGGGTTGCAAGTTGCCGT
CD163 F:	5'-CTCTGAATGACCCCCGAGGA
CD163 R:	5'-CACGGCACTCTTGGTTTGTG
IL-1β F:	5'-CTGGTGTGTGACGTTCCCATTA
IL-1β R:	5'-CCGACAGCACGAGGCTTT
IL-6 F:	5'-GGCCTTCCCTACTTCACAAG
IL-6 R:	5'-ATTTCCACGATTTCCCAGAG
iNOS F:	5'-GCTATGGCCGCTTTGATGTG
iNOS R:	5'-TCGAACTCCAATCTCGGTGC
NOX2 F:	5'-GGGAACTGGGCTGTGAATGA
NOX2 R:	5'-CAGTGCTGACCCAAGGAGTT
TGFβ1 F:	5'-CTCCCGTGGCTTCTAGTGC
TGFβ1 R:	5'-GCCTTAGTTTGGACAGGATCTG
Ym1 F:	5'-ACCCCTGCCTGTGTACTCACCT
Ym1 R:	5'-CACTGAACGGGGCAGGTCCAAA
TLR2 F:	5'-GCAAACGCTGTTCTGCTCAG
TLR2 R:	5'-AGGCGTCTCCCTCTATTGTATT
TLR3 F:	5'-TTGTCTTCTGCACGAACCTG
TLR3 R:	5'-GGCAACGCAAGGATTTTATT
TLR4 F:	5'-GCCTTTCAGGGAATTAAGCTCC
TLR4 R:	5'-AGATCAACCGATGGACGTGTAA
TLR7 F:	5'-ATGTGGACACGGAAGAGACAA
TLR7 R:	5'-GGTAAGGGTAAGATTGGTGGTG
FasL F:	5'-TTGAAAAGCAAATAGCCAACCC
FasL R:	5'-CACTCCAGAGATCAGAGCGG
FasR F:	5'-GCACCCTGACCCAGAATACC
FasR R:	5'-GTTCCATGTTCACACGAGTC
NeuN F:	5'-AACTTATGGAGCGGTCGTGT
NeuN R:	5'-GGCCGATGGTGTGATGGTAA
MAP2 F:	5'-GAGGAAGCAGCAAGTGGTGA
MAP2 R:	5'-GGGAGGATGGAGGAAGGTCT
DCX F:	5'-GACACCATGTGCTTAGGGCT
DCX R:	5'-ATTTGGGCAGTTTTCCCCCT
GFAP F:	5'-TCCTGGAACAGCAAAACAAG
GFAP R:	5'-CAGCCTCAGGTTGGTTTCAT
S100β F:	5'-CTAGGCATTCCCGTGAGCTG
S100β R:	5'-ATGAGCAACCTCTTCGGGTG
MBP F:	5'-CCCTCACGTTATTGTGGCGA
MBP R:	5'-AGACCTTCCAAAGAGCCCCA
CNP F:	5'-GACATAGTACCCGCAAAGGC
CNP R:	5'-AAGAGCTTGGGCAGGAATGT
Pecam1 F:	5'-GCATCGGCAAAGTGGTCAAG
Pecam1 R:	5'-TTGCTGGGTCATTGGAGGTC
ICAM2 F:	5'-ACTGGCACAGAGGAGATTGTG
ICAM2 R:	5'-AGGCTCCAGCAAGCAAAAGA
CD200 F:	5'-CCGAGAAGCTGGTGTCTAGC
CD200 R:	5'-ACCACTTCCACTTGAGCTGT
CX_3_CL1 F:	5'- CAACTTCCGAGGCACAGGAT
CX_3_CL1 R:	5'- CCAAACGGTGGTGGAGATGT
c-Fos F:	5'-TGGCACTAGAGACGGACAGA
c-Fos R:	5'-TTTCAACGCCGACTACGAGG
EGR1 F:	5'-GCGATGGTGGAGACGAGTTA
EGR1 R:	5'-AGAGGTCGGAGGATTGGTCA
c-Jun F:	5'-TGGGCACATCACCACTACAC
c-Jun R:	5'-TGACACTGGGAAGCGTGTTC
Arc F:	5'-CCAAGCCCCAGCTCCAATTA
Arc R:	5'-CCTACACACCCTATGCCAGC
C3 F:	5'-CAGGACGTGAGAGTCGATGG
C3 R:	5'-CTCTGCCTATGCTGCCTTCA
C1qA F:	5'-GAAGGGCGTGAAAGGCAATC
C1qA R:	5'-CAAGCGTCATTGGGTTCTGC

### Protein isolation and ELISAs

Total protein was extracted from frozen half-brains by homogenization in cold lysis buffer (20 mM Tris, 0.25 M sucrose, 2 mM EDTA, 10 mM EGTA, 1% Triton X-100 plus 1 tablet of Complete ULTRA protease inhibitor cocktail [Sigma, St. Louis, MO] per 10 mL solution). Homogenates were centrifuged at 100,000*g* for 40 min, supernatant was collected, and protein levels determined using the BCA protein assay reagent kit (PIERCE, Milwaukee, WI). Levels of TNFα, Ccl2, IL-1ra, and IL-4 were measured using commercial enzyme-linked immunosorbent assay (ELISA) kits from R&D Systems (Minneapolis, MN), as per the manufacturer’s instructions.

### Perfusion and brain tissue preparation

Mice were anesthetized with sodium pentobarbital (100 mg/kg, i.p.) and transcardially perfused with 0.1 M phosphate-buffered saline (PBS, pH 7.4). The brains used for RT-PCR or ELISAs were extracted and immediately frozen in liquid nitrogen. The brains used for immunohistochemistry were perfused with 4.0% paraformaldehyde in PBS, extracted, post-fixed in 4.0% paraformaldehyde/PBS solution overnight, and then placed in a 30% sucrose solution in PBS for a few days. Brain tissue was sectioned coronally at a thickness of 40 μm on a sliding microtome (MICROM HM450; ThermoScientific, Austin, TX). Sections were sequentially collected into well plates and stored at −20 °C in a cryoprotectant solution consisting of 30% glycol/30% ethylene glycol in PBS for immunohistochemistry.

### Immunohistochemistry

Free-floating sections were washed in 0.1 M PBS, incubated in 0.3% H_2_O_2_ for 30 min, washed again in PBS, and blocked for 1 h at room temperature in 0.25% Triton-X100/5% normal serum (MP Biomedicals, Solon, OH, Cat. # 19135680). Sections were transferred directly from the block to primary antibody (rabbit anti-Iba1, WAKO, Japan) diluted in blocking solution and were incubated overnight at 4 °C. Sections were then washed in PBS, incubated for 1 h in biotinylated secondary antibody (1:200; Vector Laboratories, Burlingame, CA), and washed and incubated for 1 h in avidin–biotin complex solution (Vectastain ABC Kit, Vector Laboratories, Burlingame, CA, Cat. # PK6100). The chromogen, nickel-enhanced diaminobenzidine (Sigma-Aldrich, St. Louis, MO, Cat. # D5637), was used to visualize immunoreactivity. Tissue was mounted onto slides, dehydrated, and coverslipped.

### Rotarod behavioral experiments

Rotarod testing was performed as previously described [30] and consisted of walking on a rotarod apparatus (Ugo Basile, Italy, Mouse Rota-rod, 47600) set at a fixed speed of 16 rotations-per-minute (rpm). Each mouse underwent training one day prior to experimental testing. Training consisted of the mouse remaining on the rod for three consecutive trials of 180 s. Mice that did not pass this criterion within 18 trials were not included in subsequent testing. The next day, mice performed a baseline test to ensure they could remain on the rotarod for 180 s. Mice were then injected i.p. with either vehicle or the recombinant IL-1ra drug, Kineret (obtained from the pharmacy at UNC Hospitals) at a dose of either 100 or 300 mg/kg. Thirty minutes later, mice underwent another rotarod test to ensure there were no effects of IL-1ra on motor activity. Mice were then injected i.p. with ethanol (2.0 g/kg, 20% *v*/*v*) and tested on the rotarod at 2, 5, 8, 14, 20, and every subsequent 10 min until 110 min had passed. The time the mice remained on the rotarod was recorded.

Rotarod testing was performed on another group of mice, except that these mice received either control chow or PLX5622 chow for 1 week prior to testing, a treatment time previously shown to result in substantial microglial depletion [31]. These mice were otherwise trained and tested as described above.

### Behavioral assessment of intoxication following different doses of acute binge ethanol

To determine the level of intoxication in mice receiving the 4.5 or 6.0 g/kg doses of ethanol, behavioral assessments of movement and pain response were performed each hour post-gavage, similar to previous studies [32]. For assessing movement, a complete absence of movement other than breathing was recorded as “No Activity.” Ataxic movement of the head or limbs or impaired ambulation was recorded as “Impaired Activity.” Movement that appeared indistinguishable from a sober control mouse was recorded as “Full Activity.” For pain assessment, each hindpaw was pinched. A complete absence of a response was recorded as “No response.” Slight flinching or movement following any pinch was recorded as “Weak Response.” Full-paw withdrawal for any pinch was recorded as “Full Response.”

### Statistical analyses

The Statistical Package for the Social Sciences (SPSS; Chicago, IL) was used for all statistical analyses. Data from time course experiments was analyzed via ANOVA with Tukey’s post hoc test for multiple comparisons. The data from dose-response experiments was analyzed via ANOVA with Dunnett’s post hoc test compared to controls. Data from microglial depletion experiments was analyzed using a two-by-two ANOVA with significant interactions being further investigated using Tukey’s post hoc test for multiple comparisons. Comparison of two means was analyzed using Student’s *t*- test. All values are reported as mean ± SEM, and significance was defined at a level of *p* ≤ 0.05.

## Results

### Acute binge ethanol biphasically and dose dependently alters microglial gene expression in vivo

**Fig. 1:**
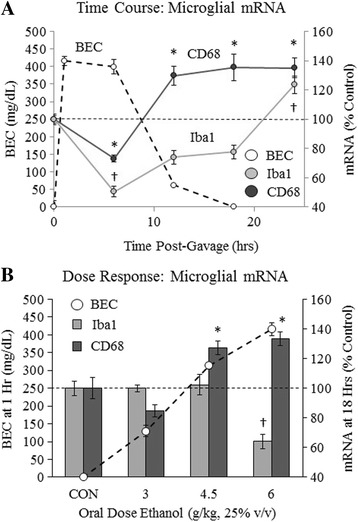
Acute binge ethanol biphasically and dose dependently changes microglial gene expression. **a** Time course of blood alcohol concentrations and brain microglial mRNA following acute binge ethanol: Mice were gavaged with ethanol (6 g/kg, 25% *v*/*v*) and tail blood was collected at 1, 6, 12, and 18 h to determine BACs (*dashed line*—*left axis*). Brain mRNA was collected at 0, 6, 12, 18, or 24 h and microglial Iba1 and CD68 mRNA levels were measured (*solid lines*—*right axis*). Note that Iba1 and CD68 mRNA decreased when BACs were high and increased when BACs dropped. **b** Ethanol dose-response curve: Mice were gavaged with ethanol (3, 4.5, or 6 g/kg, 25% *v*/*v*) or water. Tail blood was collected 1 h post-gavage to assess BACs (*dashed line*—*right axis*). At 18 h post-gavage, brain mRNA was collected and microglial Iba1 and CD68 mRNA levels were measured (*light and dark gray bars*, *left axis*). Ethanol dose dependently altered Iba1 and CD68 mRNA. Data are presented as mean ± SEM. *p < 0.05 for CD68, ^†^ *p* < 0.05 for Iba1. *n* = 5–7/group

Previous studies have found that chronic ethanol increases microglial markers in vivo [33], and that acute ethanol has varying effects over time on human monocytes in vitro [19]. However, few studies have investigated the effects of acute ethanol on microglia in vivo. Furthermore, it is unclear what dose of ethanol is required to impact microglia acutely in vivo. We therefore performed time course and dose-response experiments with acute binge ethanol in vivo. Mice were treated intragastrically with various doses of ethanol (3, 4.5, and 6 g/kg), and a time course was done with the highest dose (6 g/kg). Brain mRNA levels of commonly studied microglial markers, Iba1 and CD68, were assessed. Only high doses of ethanol (i.e., 4.5 and 6 g/kg) altered Iba1 and CD68 mRNA (Fig. 1). Although these doses yielded high BACs (≥300 mg/dL), no mice died (Additional file 2: Figure S2). To assess the level of intoxication in mice receiving these doses, behavioral assessments of movement and pain response were performed each hour post-gavage, similar to previous studies [32]. There was a transient decrease in movement and pain response while BACs were high, with normal behavior returning as BACs approached zero (Additional file 2 Figure S2). For the time course, tail blood was collected at 1, 6, 12, and 18 h post-gavage. BACs were approximately 400 mg/dL at 1 h and decreased to 0 mg/dL by 18 h (Fig. 1a). Interestingly, a biphasic effect on Iba1 mRNA levels was observed, with a 50% decrease (*p* < 0.05) at 6 h, followed by a 23% increase at 24 h (*p* < 0.05) (Fig. 1a). CD68 mRNA showed a similar biphasic response, with a 27% decrease (*p* < 0.05) at 6 h and a 30% (*p* < 0.05), 36% (*p* < 0.05), and 35% (*p* < 0.05) increase at 12, 18, and 24 h, respectively (Fig. 1a). Gavage with water did not change brain CD68 or Iba1 mRNA over time (Additional file 3: Figure S3). For the dose-response studies, tail blood was collected at 1 h post-gavage. Increasing doses of ethanol caused a proportional increase in BACs at 1 h (Fig. 1b). Mice were sacrificed 18 h post-gavage, a point at which time course data showed elevated microglial CD68 mRNA. The 4.5 g/kg dose increased CD68 mRNA 27% (*p* < 0.05) but did not change Iba1 mRNA (Fig. 1b). The 6 g/kg dose increased CD68 mRNA 33% (*p* < 0.05), while Iba1 mRNA was still decreased by 36% (*p* < 0.05) at this 18-h time point (Fig. 1b). These results find that acute binge ethanol biphasically changes microglial gene expression in vivo, with initial decreases during intoxication and later increases during withdrawal. Furthermore, these changes only happen at high doses of acute ethanol.

### Acute binge ethanol withdrawal dose dependently increases brain pro-inflammatory cytokine expression in vivo

**Fig. 2:**
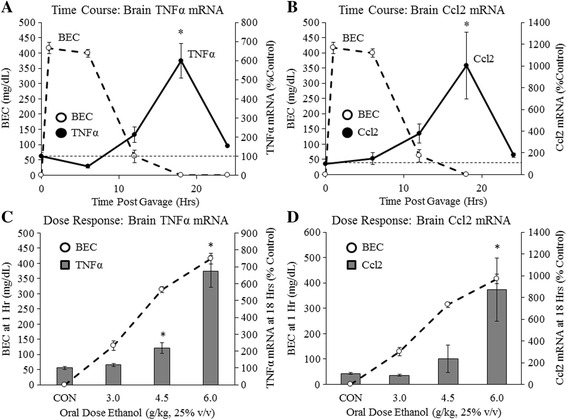
Acute binge ethanol withdrawal increases brain pro-inflammatory gene expression. **a**, **b** Time course of blood alcohol concentrations and brain TNFα and Ccl2 mRNA following acute binge ethanol: Mice were gavaged with ethanol (6 g/kg, 25% *v*/*v*) and tail blood was collected at 1, 6, 12, and 18 h to determine BACs (*dashed line*—*left axis*). Brain mRNA was collected at 0, 6, 12, 18, or 24 h and TNFα mRNA and Ccl2 mRNA were assessed (*solid line*—*right axis*). Note that TNFα and Ccl2 mRNA increased during withdrawal when BACs dropped. **c**, **d** Ethanol dose-response curve: Mice were gavaged with ethanol (3, 4.5, or 6 g/kg, 25% *v*/*v*) or water. Tail blood was collected 1 h post-gavage to assess BACs (*dashed line*—*right axis*). At 18 h post-gavage, brain mRNA was collected and TNFα and Ccl2 transcript levels were assessed (*gray bars*, *left axis*). Data are presented as mean ± SEM. *p < 0.05. *n* = 5–8/group

Increased microglial markers suggest microglial activation but do not indicate the functional changes occurring in microglia. Microglia can adopt both pro- and anti-inflammatory activation states [16, 17]. We therefore sought to assess changes in pro-inflammatory cytokine expression following acute binge ethanol. Time course experiments were performed as described above. We measured the brain expression of TNFα and Ccl2, key pro-inflammatory cytokines. TNFα expression decreased by 54% at 6 h post-treatment and increased dramatically during withdrawal, peaking at 600% (*p* < 0.05) at 18 h (Fig. 2a). Ccl2 showed a similar pattern of expression and increased during ethanol withdrawal, peaking at 1002% (*p* < 0.05) at 18 h (Fig. 2b). Gavage with water did not significantly increase TNFα or Ccl2 mRNA at any time point (Additional file 4: Figure S4). These marked changes in mRNA levels were accompanied by increased brain TNFα and Ccl2 protein levels at 18 h post-gavage. TNFα protein increased 345%, while Ccl2 increased from less than 1 pg/mL to approximately 17 pg/mL as determined by ELISAs (Additional file 5: Figure S5). These results find that acute binge ethanol withdrawal increases brain TNFα and Ccl2 mRNA and protein during withdrawal, similar to the microglial markers.

In order to determine the dose of acute binge ethanol required to impact pro-inflammatory cytokine expression, we performed in vivo dose-response curves as described above. The 3 g/kg dose of ethanol did not change TNFα or Ccl2 at the 18-h time point. The 4.5 g/kg dose of ethanol increased TNFα 218% (*p* < 0.05) and Ccl2 236% at 18 h post-gavage (Fig. 2c, d). TNFα and Ccl2 expression increased exponentially to 672% (*p* < 0.05) and 873% (*p* < 0.05), respectively, with the 6 g/kg dose (Fig. 2c, d). These results find that acute binge ethanol dose dependently increases brain pro-inflammatory cytokine expression during withdrawal beginning at high doses.

### Acute binge ethanol withdrawal dose dependently increases brain anti-inflammatory cytokine expression in vivo

**Fig. 3:**
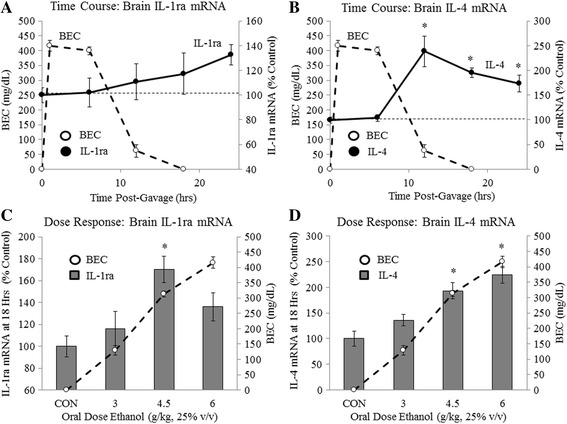
Acute binge ethanol withdrawal increases brain anti-inflammatory gene expression. **a**, **b** Time course of blood alcohol concentrations and brain IL-1ra and IL-4 mRNA following acute binge ethanol: Mice were gavaged with ethanol (6 g/kg, 25% *v*/*v*) and tail blood was collected at 1, 6, 12, and 18 h to determine BACs (*dashed line*—*left axis*). Brain mRNA was collected at 0, 6, 12, 18, or 24 h and IL-1ra and IL-4 mRNA were assessed (*solid line*—*right axis*). **c**, **d** Ethanol dose-response curve: Mice were gavaged with ethanol (3, 4.5, or 6 g/kg, 25% *v*/*v*) or water. Tail blood was collected 1 h post-gavage to assess BACs (*dashed line*—*right axis*). At 18 h post-gavage, brain mRNA was collected and IL-1ra and IL-4 mRNA were assessed (*gray bars*, *left axis*). Data are presented as mean ± SEM. *p < 0.05. *n* = 5–8/group

Microglia often react to insults in a complex and dynamic manner consisting of both pro-inflammatory and anti-inflammatory responses. We therefore assessed changes in brain anti-inflammatory gene expression following acute binge ethanol. Mice were treated with acute binge ethanol as described above. We measured expression of IL-1ra and IL-4, key anti-inflammatory cytokines. IL-1ra expression increased modestly to 132% at 24 h after ethanol treatment (Fig. 3a). Expression of IL-4 peaked at 238% (*p* < 0.05) at 12 h and decreased to 174% (*p* < 0.05) by 24 h (Fig. 3b). Gavage with water did not significantly increase IL-1ra or IL-4 mRNA at these time points (Additional file 4: Figure S4). Similar changes in brain IL-1ra and IL-4 protein were observed 18 h post-gavage. While IL-1ra protein was unchanged, IL-4 protein increased approximately ninefold as determined by ELISAs (Additional file 5: Figure S5). These results find that acute binge ethanol increases brain IL-4 mRNA and protein during withdrawal.

In order to determine the dose of acute binge ethanol required to impact anti-inflammatory cytokine expression, we performed in vivo dose-response curves as described above. The 3 g/kg dose of ethanol did not change IL-1ra or IL-4 at the 18-h time point. The 4.5 g/kg dose of ethanol increased IL-1ra 170% (*p* < 0.05) and IL-4 193% (*p* < 0.05) at 18 h post-gavage (Fig. 3c, d). IL-1ra expression decreased to 136% with the 6 g/kg dose, while IL-4 expression increased to 224% (*p* < 0.05) (Fig. 3c, d). These data find that acute binge ethanol dose dependently alters brain anti-inflammatory cytokine expression during withdrawal.

### Acute ethanol exposure alters microglial cytokine expression in vitro

**Fig. 4:**
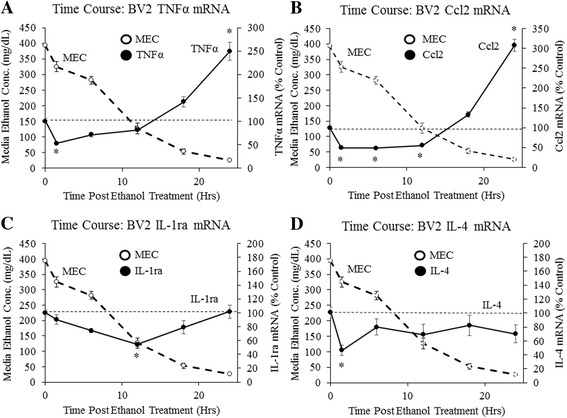
Acute ethanol changes BV2 microglial cytokine gene expression in vitro. Time course of cytokine expression in ethanol-treated BV2 cells: Microglia-like BV2 cells were treated with ethanol (85 mM) and the ethanol evaporated over time. Media ethanol concentrations (MECs) were measured (*dashed line*—*left axis*) and mRNA was collected from the BV2 cells. mRNA levels of **a** TNFα **b** Ccl2 **c** IL-1ra, and **d** IL-4 in ethanol-treated cells were normalized to mRNA levels in PBS-treated control cells (*solid line*—*right axis*). Data are presented as mean ± SEM. *p < 0.05. *n* = 4–6/group

Ethanol-induced changes in neuroimmune gene expression in vivo could be due to direct actions of ethanol on microglia or due to immune signaling across other brain cell types, such as endothelial cells, astrocytes, and even neurons [34–36]. To determine whether ethanol exposure and withdrawal can directly alter microglial gene expression, cultured BV2 microglia-like cells were treated with ethanol in vitro. Cells were treated with ethanol, but without vaporized ethanol in the incubation chamber, allowing the ethanol to evaporate from the media and simulate the ethanol clearance that occurs in vivo. Media ethanol concentrations were measured and BV2 mRNA was collected over time. Ethanol concentrations decreased from an initial concentration of approximately 400 mg/dL to nearly 0 mg/dL over 24 h (Fig. 4). Gene expression in ethanol-treated BV2 cells was normalized to gene expression in PBS-treated BV2 cells at each time point. This was done to normalize gene expression changes over time (Additional file 6: Figure S6). Ethanol treatment initially decreased TNFα mRNA 47% (*p* < 0.05) at 1.5 h post-treatment, but later increased TNFα mRNA to 250% (*p* < 0.05) of controls 24 h post-treatment (Fig. 4a). Ccl2 mRNA showed a similar pattern, with an initial decrease in expression through 12 h of treatment followed by a 312% (*p* < 0.05) increase at 24 h post-treatment (Fig. 4b). Ethanol also decreased IL-1ra expression 45% (*p* < 0.05) at 12 h and decreased IL-4 expression 53% (*p* < 0.05) at 1.5 h (Fig. 4c, d). To determine whether decreases in BV2 gene expression were due to cell death, we used the vital stain Trypan blue to determine cell viability. Results showed no changes in BV2 viability at multiple time points of post-ethanol treatment (Additional file 7: Figure S7). To determine whether increased pro-inflammatory gene expression was dependent on ethanol evaporation, identical experiments were performed, except ethanol was vaporized into the incubator chamber to keep MECs constant (Additional file 8: Figure S8). After 24 h of continuous ethanol exposure, BV2 TNFα mRNA was unchanged, while Ccl2 mRNA was decreased 17% (*p* < 0.05) (Additional file 8: Figure S8), consistent with ethanol evaporation being required for increased pro-inflammatory gene expression. These results find that ethanol treatment biphasically alters BV2 microglia-like gene expression in vitro, with increased pro-inflammatory gene expression occurring after ethanol clearance, and consistent with ethanol having direct effects on microglia.

### PLX5622 decreases expression of microglial genes and alters expression of some neuroimmune genes in vivo

**Fig. 5:**
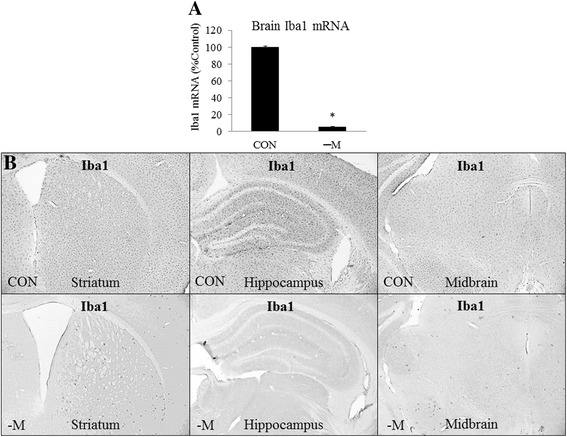
Treatment with CSF1R inhibitor PLX5622 depletes microglia from the brains of mice. C57BL/6J mice received chow containing 1200 mg/kg PLX5622 for 3 weeks. **a** RT-PCR on whole brain mRNA showed a 94% reduction in Iba1 mRNA, indicating substantial depletion of microglia. Data are represented as mean ± SEM. *p < 0.05, Student’s *t* test, *n* = 6/group. **b** Immunohistochemical staining for Iba1 confirmed depletion of microglia across the brain. Illustrations show a section of the striatum, hippocampus and midbrain

**Fig. 6:**
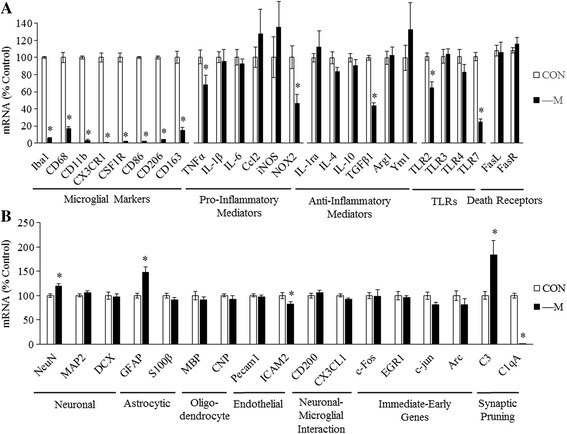
Treatment with CSF1R inhibitor PLX5622 alters brain gene expression. Mice received chow containing PLX5622 for 3 weeks and mRNA was isolated from whole brain. **a** RT-PCR showed marked reduction in several microglial transcripts, indicating substantial microglial depletion. Other neuroimmune transcripts were also assessed. **b** The effects of PLX5622 on non-microglial transcripts were also assessed. Note that PLX5622 had little to no effect on neuronal, oligodendrocyte, or endothelial transcripts, but increased the astrocyte marker GFAP. Also note the marked changes in synaptic pruning genes, C3 and C1qA. Data are represented as mean ± SEM. *p < 0.05, Student’s *t* test, *n* = 6/group

To further understand which gene expression changes are due to microglia, we sought to deplete microglia from the brain and then administer acute binge ethanol. This would allow us to assess the contribution of microglial and non-microglial cells to the brain response to ethanol. Microglial depletion was performed by administering the CSF1R inhibitor PLX5622 to C57BL/6J mice for 3 weeks, a duration previously shown to result in maximal microglial depletion [28]. We first characterized the effects of PLX5622 treatment on microglial and neuroimmune gene expression. RT-PCR and immunohistochemistry for the microglial marker Iba1 found that PLX5622 reduced Iba1 mRNA 94% (*p* < 0.05) (Fig. 5a) and almost completely eliminated Iba1+ cells (Fig. 5b), consistent with PLX5622 depleting microglia from the brain. Expression of several other microglial markers (CD68, CD11b, CX_3_CR1, etc.) was reduced by 80% or more by PLX5622 (*p* < 0.05) (Fig. 6a), further suggesting substantial microglial depletion. Expression of pro-inflammatory genes, anti-inflammatory genes, and many other genes was also assessed. Interestingly, microglial depletion decreased basal expression of TNFα 32% (*p* < 0.05) and NOX2 54% (*p* < 0.05) (Fig. 6a) but did not decrease basal expression of IL-1β, IL-6, or iNOS, suggesting these genes may be expressed in other cell types. PLX5622 decreased expression of TGFβ1 56% (*p* < 0.05) but did not decrease expression of other examined anti-inflammatory genes. We also examined expression of multiple Toll-like receptors (TLRs)—a group of innate immune receptors. PLX5622 decreased brain expression of TLR2 36% (*p* < 0.05) and TLR7 76% (*p* < 0.05) but surprisingly did not decrease expression of TLR3 and TLR4 (Fig. 6a). This suggests these neuroimmune genes may be expressed in other CNS cell types. Furthermore, expression of genes encoding death receptors—receptors that contribute to apoptosis—and death receptor ligands, such as FasL and FasR, were unchanged with microglial depletion. These results suggest that PLX5622 depletes microglia, but does not alter expression of many genes associated with neuroimmune signaling.

In order to investigate the specificity of PLX5622 for microglia and potential effects on other brain cell types, we assessed the expression of several other genes. Microglial depletion caused no change in expression of the neuronal marker MAP2 or neuroprogenitor marker DCX. However, there was a small, but statistically significant increase in expression of neuronal marker NeuN (Fig. 6b) and a 48% increase in astrocyte marker GFAP (*p* < 0.05). There was no effect on astrocytic S100β expression (Fig. 6b). Oligodendrocyte markers myelin basic protein (MBP) and 2′,3′-cyclic-nucleotide 3′-phosphodiesterase (CNP) were not changed by microglial depletion (Fig. 6b). There was a small, but statistically significant 17% decrease in endothelial marker ICAM2, but no change in endothelial marker Pecam1 (Fig. 6b). We also examined expression of the neuronal-microglial interaction genes, CD200 and CX_3_CL1 (also known as fractalkine). CD200 and CX_3_CL1 are expressed on neurons and interact with the CD200 receptor and CX_3_CR1 located on microglia to regulate their activity [37]. Interestingly, microglial depletion did not change neuronally expressed CD200 and CX_3_CL1 (Fig. 6b), despite microglial CX_3_CR1 being markedly decreased. Since microglia play a role in synaptic elimination and synaptic plasticity [38], we also assessed the expression of C3 and C1qA, complement factors involved in synaptic pruning, as well as four immediate-early genes (IEGs), genes that are induced following neuronal activation. Microglial depletion increased C3 expression 84% (*p* < 0.05) and decreased C1qA expression to 1% (*p* < 0.05). No significant changes in either c-Fos, EGR1, c-jun, or Arc were found (Fig. 6b). These results find that microglial depletion has little effect on basal expression of several markers of other CNS cell types.

### Microglial depletion alters the brain neuroimmune response to acute binge ethanol withdrawal

**Fig. 7:**
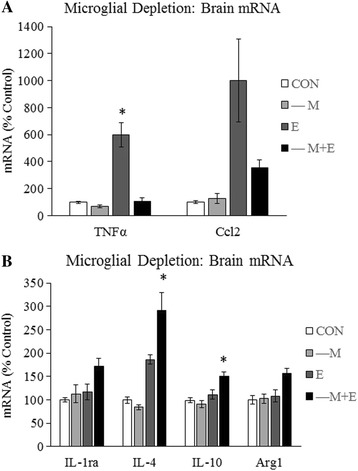
Microglial depletion alters the neuroimmune response to acute binge ethanol withdrawal. Microglia-depleted mice were gavaged with either ethanol (6 g/kg, 25% *v*/*v*) or water and sacrificed 18 h later. **a** Levels of pro-inflammatory TNFα and Ccl2 transcripts were determined in whole brain mRNA. **b** Levels of anti-inflammatory IL-1ra, IL-10, IL-4, and Arg1 were determined in whole brain mRNA. Data are represented as mean ± SEM. *p < 0.05 Tukey’s post hoc test compared to all other groups. *n* = 6–8/group

To determine the role of microglia in the brain response to acute binge ethanol withdrawal, microglia were depleted from the brains of mice as described above. Microglia-depleted mice were gavaged with ethanol and brain mRNA was isolated 18 h post-treatment, a time when expression of microglial and cytokine genes was maximal. Expression of several genes was assessed via RT-PCR (Table 2). Microglial depletion completely blocked ethanol withdrawal-induced TNFα expression (Fig. 7a), reducing TNFα mRNA from about 600 to 104% (*p* < 0.05). Microglial depletion partially blocked ethanol withdrawal-induced Ccl2 expression, decreasing Ccl2 mRNA from approximately 1000 to 355% (Fig. 7a). Interestingly, microglial depletion enhanced the ethanol withdrawal response of several anti-inflammatory genes. Withdrawal-induced IL-1ra was increased from 117 to 173% with microglial depletion (Fig. 7b). Microglial depletion also enhanced the ethanol withdrawal response of anti-inflammatory cytokines IL-4 and IL-10 to 291% (*p* < 0.05) and 151% (*p* < 0.05), respectively (Fig. 7b). These results find that microglial depletion blunts the acute binge ethanol withdrawal pro-inflammatory gene response and enhances the anti-inflammatory gene response.

**Table 2:** Effects of acute binge ethanol and/or microglial depletion on brain gene expression

	CON	–M	E	–M + E
Microglial markers				
Iba1^a, b, c^	100 ± 1^A^	6 ± 0.4^B^	68 ± 3^C^	6 ± 1^B^
CD11b^a, b, c^	100 ± 2^A^	3 ± 1^B^	57 ± 2^C^	5 ± 1^B^
CD45^a^	100 ± 7	52 ± 7	121 ± 15	69 ± 5
MHCII	100 ± 8	68 ± 22	67 ± 4	48 ± 15
M1 microglial markers				
CD68^a, b^	100 ± 6	17 ± 2	136 ± 9	42 ± 4
CD86^a, b, c^	100 ± 1^A^	2 ± 0.3^B^	127 ± 6^C^	5 ± 2^B^
iNOS^a, b, c^	100 ± 24^A^	135 ± 37^A^	170 ± 37^A^	812 ± 145^B^
NOX2^a^	100 ± 13	46 ± 11	137 ± 14	51 ± 10
M2 microglial markers				
CD206^a^	100 ± 2	4 ± 0.2	91 ± 5	5 ± 0.3
CD163^a, b^	100 ± 7	15 ± 3	127 ± 11	30 ± 3
Arg1^a, b^	100 ± 8	103 ± 10	108 ± 13	157 ± 11
Ym1	100 ± 15	133 ± 32	194 ± 36	197 ± 57
Pro-inflammatory cytokines				
IL-1β^b^	100 ± 6	95 ± 14	143 ± 17	174 ± 16
TNFα^a, b, c^	100 ± 8^A^	68 ± 11^A^	600 ± 90^B^	104 ± 26^A^
IL-6^b^	100 ± 9	92 ± 6	131 ± 13	164 ± 9
Ccl2^b^	100 ± 12	127 ± 36	1002 ± 308	355 ± 60
Anti-inflammatory cytokines				
IL-10^b, c^	100 ± 5^A^	91 ± 7^A^	112 ± 11^A^	152 ± 9^B^
IL-4^b, c^	100 ± 7^A, B^	84 ± 5^A^	186 ± 10^B^	291 ± 40^C^
IL-1ra^a, b^	100 ± 5	113 ± 19	117 ± 17	173 ± 16
TGF-β1^a^	100 ± 3	44 ± 3	107 ± 11	58 ± 8
CNS cell types				
NeuN^a^	100 ± 4	120 ± 5	108 ± 5	125 ± 10
MAP2	100 ± 3	106 ± 4	108 ± 4	102 ± 5
DCX^b^	100 ± 7	97 ± 7	84 ± 3	83 ± 6
GFAP^a^	100 ± 5	148 ± 11	111 ± 11	145 ± 10
S100β	100 ± 5	91 ± 5	91 ± 4	95 ± 6
CNP	100 ± 3	93 ± 7	86 ± 2	92 ± 7
MBP	100 ± 9	91 ± 6	84 ± 7	85 ± 5
ICAM2^b, c^	100 ± 6^A^	83 ± 5^A^	108 ± 11^A^	144 ± 8^B^
Pecam1	100 ± 3	97 ± 4	103 ± 9	110 ± 7
Toll-like receptors				
TLR2^a, b^	100 ± 4	64 ± 7	133 ± 8	94 ± 7
TLR3	100 ± 8	103 ± 6	99 ± 3	100 ± 5
TLR4^b^	100 ± 8	82 ± 9	137 ± 11	125 ± 11
TLR7^a, b, c^	100 ± 5^A^	24 ± 4^B^	152 ± 6^C^	38 ± 3^B^
Neuron-microglia signaling				
CX3CL1^a, b, c^	100 ± 3^A^	93 ± 2^A^	96 ± 4^A^	75 ± 3^B^
CX3CR1^a^	100 ± 5	1 ± 0.1	112 ± 15	4 ± 1
CD200	100 ± 5	106 ± 5	102 ± 3	104 ± 3
CD200R1	100 ± 16	70 ± 9	105 ± 16	91 ± 8
CSF1	100 ± 2	120 ± 5	113 ± 5	104 ± 5
CSF1R^a^	100 ± 5	2 ± 0.3	91 ± 4	4 ± 1
Neurotrophins				
BDNF^a, c^	100 ± 4^A, B^	101 ± 4^A, B^	113 ± 6^A^	86 ± 5^B^
NGF	100 ± 6	103 ± 3	96 ± 4	107 ± 4
NT3	100 ± 7	111 ± 9	96 ± 6	117 ± 10
NT4/5^a, b^	100 ± 6	115 ± 15	131 ± 10	176 ± 17
Cytokine receptors				
IL-1R1^a, b^	100 ± 4	113 ± 8	130 ± 6	189 ± 20
TNFR1^b^	100 ± 2	101 ± 5	131 ± 4	120 ± 6
TNFR2^a, b^	100 ± 5	55 ± 2	124 ± 5	70 ± 6
CCR2	100 ± 11	110 ± 21	131 ± 22	144 ± 13
Immediate-early genes				
c-Fos	100 ± 6	99 ± 13	155 ± 26	123 ± 20
EGR1	100 ± 9	96 ± 4	121 ± 8	92 ± 13
Arc	100 ± 10	81 ± 13	105 ± 17	75 ± 19
c-Jun^b, c^	100 ± 7^A, B^	81 ± 5^A^	105 ± 5^A, B^	116 ± 8^B^
Apoptosis pathways				
FasL^a, b, c^	100 ± 6^A^	98 ± 11^A^	121 ± 8^A^	186 ± 20^B^
FasR^b^	100 ± 3	107 ± 7	146 ± 17	210 ± 25
TL1A^b^	100 ± 9	88 ± 8	127 ± 25	157 ± 12
DR3^a^	100 ± 5	71 ± 7	91 ± 9	55 ± 9
TRAIL^b^	100 ± 5	108 ± 5	88 ± 7	67 ± 13
DR5^a^	100 ± 6	106 ± 5	91 ± 6	139 ± 17
LTα^a, b, c^	100 ± 5^A^	95 ± 10^A^	120 ± 19^A^	233 ± 25^B^
FADD	100 ± 3	98 ± 5	99 ± 4	114 ± 14
Caspase-3	100 ± 4	112 ± 4	112 ± 4	130 ± 12
Clock genes				
Per1^b^	100 ± 3	111 ± 8	155 ± 6	185 ± 41
Per2^b, c^	100 ± 8^A^	123 ± 5^A, B^	202 ± 7^C^	147 ± 12^B^
BMAL1	100 ± 5	95 ± 5	104 ± 4	110 ± 6
Neurotransmitter systems				
CRF^a^	100 ± 8	87 ± 7	93 ± 6	73 ± 7
DAT	100 ± 5	98 ± 7	92 ± 4	100 ± 4
GluR1^b^	100 ± 2	101 ± 4	123 ± 3	121 ± 10
NPY	100 ± 2	95 ± 4	96 ± 4	96 ± 3
Other				
Glucocorticoid Receptor	100 ± 5	99 ± 3	96 ± 3	90 ± 6
HMGB1	100 ± 4	103 ± 7	101 ± 4	109 ± 4
COX2^b^	100 ± 7	106 ± 5	140 ± 7	151 ± 9
MMP9^b^	100 ± 2	106 ± 7	125 ± 6	133 ± 8
IL-12	100 ± 5	112 ± 7	99 ± 8	101 ± 6
IL-34	100 ± 7	101 ± 6	96 ± 7	262 ± 80
NLRP3^a, b^	100 ± 3	27 ± 4	159 ± 22	57 ± 6
Nogo	100 ± 4	114 ± 6	124 ± 5	116 ± 3

Values are for mRNA levels set to 100% of controls. Control or microglia-depleted mice were gavaged with either water or ethanol (6 g/kg, 25% *v*/*v*) and sacrificed 18 h post-treatment

A, B, C, D = means with different letters are significantly different (*p* < 0.05, Tukey’s post hoc test) from each other. Means with the same letters are not significantly different from each other. Only means with significant interactions are labeled with letters. Data represent mean ± SEM

*CON* column represents water-treated control mice, —*M, * microglia-depleted mice, *E, * ethanol-treated mice, —*M* + *E, * microglia-depleted and ethanol-treated mice

^a^Main effect of microglial depletion

^b^Main effect of ethanol treatment

^c^Interaction

Previous studies have implicated microglia in ethanol-induced neurodegeneration [33]. We therefore sought to examine whether microglial depletion changed ethanol induction of cell death processes. One mechanism by which ethanol can induce cell death is through death receptors—a group of cytokine receptors that play a role in apoptosis. We therefore examined the role of microglia in ethanol-induced expression of death receptor genes. Microglial depletion enhanced the ethanol response of brain LTα, TL1A, and FasL to 233 (*p* < 0.05), 157, and 186% (*p* < 0.05) of control levels, respectively (Table 2). Microglial depletion also enhanced the ethanol response of FasR to 210% of control levels (Table 2). These results find that microglial depletion enhances ethanol withdrawal-induced expression of multiple death receptor genes.

### Microglial depletion does not alter the behavioral response to acute binge ethanol

Other studies suggest that microglia mediate the behavioral effects of acute ethanol treatment, specifically ethanol-induced motor impairment [30]. We therefore sought to investigate the effects of microglial depletion on ethanol-induced motor impairment. After 1 week of PLX5622 treatment, a time when microglia are mostly depleted [28], mice were treated with acute ethanol and tested for motor impairment on the rotarod as previously described [30]. There was no effect of 1 week of microglial depletion on motor coordination at any time point after ethanol treatment (Additional file 9: Figure S9). To further test the effects of microglial/monocyte-associated pro-inflammatory IL-1β signaling on ethanol-induced motor impairment, we attempted to replicate a previous study finding that Kineret, an antagonist of IL-1β signaling, blunted ethanol-induced motor impairment [30]. Mice were treated with two different doses of Kineret, 100 and 300 mg/kg, 30 min before ethanol treatment and rotarod testing. We did not observe an effect of either dose of Kineret on ethanol-induced motor impairment (Additional file 9: Figure S9). This may be due to our use of C57BL/6 mice, whereas the previous study used Balb/c mice [30]. Overall, our results do not support the hypothesis that microglia play a role in the effects of acute ethanol-induced motor impairment.

## Discussion

**Fig. 8:**
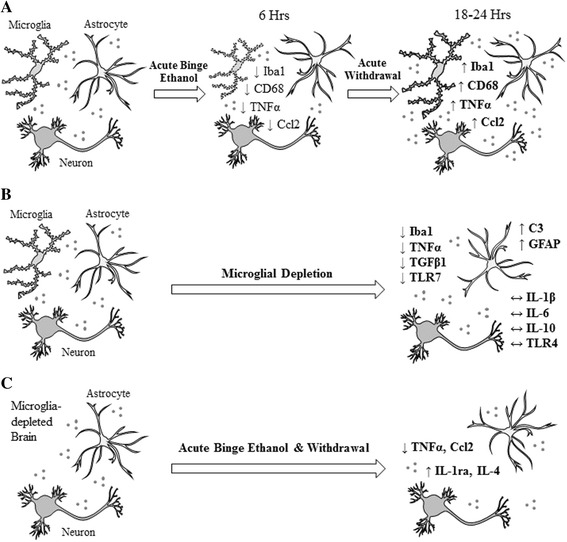
Effects of acute binge ethanol and microglial depletion on brain neuroimmune gene expression. **a** Acute binge ethanol causes biphasic changes in microglial gene expression. Various microglial genes such as Iba1 and CD68 decrease during acute intoxication. Later, during withdrawal, expression of these genes increases. These data suggest the effects of ethanol on microglia depend on time after exposure. **b** Microglial depletion alters brain gene expression. Various microglial genes, such as Iba1, decrease with microglial depletion. Other neuroimmune genes, such as TNFα or TGFβ1, also decrease. However, some neuroimmune genes, such as IL-1β, IL-6, and TLR4, do not decrease, suggesting these may be predominantly expressed in other cell types. Other genes, such as C3 and GFAP, increase with microglial depletion. **c** Microglia mediate a balance between pro- and anti-inflammatory gene expression during acute binge ethanol withdrawal. Microglial depletion blocks withdrawal-induced pro-inflammatory gene induction (TNFα and Ccl2). However, microglial depletion enhances withdrawal-induced anti-inflammatory gene induction (IL-1ra and IL-4)

In this present study, we examined the effects of acute binge ethanol on microglia and how microglial depletion changes the brain neuroimmune response to acute binge ethanol withdrawal. We report that acute binge ethanol biphasically changes microglial marker gene expression, with initial decreases during intoxication, followed by later increases during withdrawal when ethanol was gone (Fig. 8a; Additional file 10). Acute binge ethanol withdrawal dose - dependently increased neuroimmune gene expression, starting at high doses (Additional file 11). Cultured microglia-like cells showed biphasic changes in pro-inflammatory gene expression with ethanol treatment and evaporation in vitro, consistent with direct effects on microglia (Additional file 12). Also, microglial depletion reduced expression of some neuroimmune genes, while many others were unchanged, suggesting that neuroimmune genes are expressed across many brain cell types (Fig. 8b). Finally, microglial depletion blunted withdrawal-induced pro-inflammatory gene expression and enhanced withdrawal-induced anti-inflammatory gene expression (Fig. 8c; Table 2). These findings suggest microglia contribute to the impact of heavy binge alcohol on the brain, particularly during withdrawal.

These studies found that acute ethanol had dose-dependent effects on microglial and neuroimmune gene expression in vivo. Acute binge ethanol increased expression of microglial and neuroimmune genes beginning at BACs of ~300 mg/dL. To assess the level of intoxication in mice achieving these BACs, behavioral assessments of movement and pain response were performed each hour post-gavage. Behaviorally, these BACs were associated with transient reductions in movement and pain response, but no mortality. BACs of this level have also been noted in humans [2, 4, 39], suggesting these studies model brain pathology relevant to some human alcoholics. Increasing the ethanol dose further increased the expression of brain microglial and neuroimmune genes. This acute binge ethanol treatment also consistently models microglial and neuroimmune changes seen with moderate-dose, chronic ethanol treatment [10, 12]. Indeed, it seems that either high-dose, acute ethanol or moderate-dose, chronic ethanol is required to impact microglial and neuroimmune gene expression. These results are also consistent with studies in post-mortem human alcoholic brains that find increased microglial Iba1 [13, 14]. Overall, these results find that acute binge ethanol withdrawal dose dependently increases microglial and neuroimmune gene expression, with high binge doses required to increase expression acutely.

We also report that acute binge ethanol has biphasic effects on microglial gene expression in vivo. Iba1 and CD68 expression initially decreased during intoxication and later increased during withdrawal. Increased Iba1 during ethanol withdrawal is consistent with previous chronic ethanol studies [40]. However, increased CD68 protein was not observed in previous in vivo studies [18]. Since those studies report comparably high BACs, differences may be due to examining protein versus mRNA or due to different patterns of ethanol treatment. Indeed, the microglial response to stimuli can be dynamic and complex [41], and the response to ethanol likely depends on several factors, including the dose, pattern of administration, and timing after ethanol exposure. The use of different species may also account for the differences between these studies. Overall, these results show acute binge ethanol has biphasic effects on microglial gene expression in vivo (Fig. 8a).

These studies also find acute binge ethanol withdrawal increases both pro-inflammatory and anti-inflammatory gene expression in the brain. This was accompanied by increased TNFα, Ccl2, and IL-4 protein. Our results are consistent with previous studies showing acute ethanol increased brain TNFα and Ccl2 gene expression in mice [12]. Our results add to these previous observations by defining the time course of acute ethanol-induced pro-inflammatory gene expression. Specifically, it is during withdrawal that acute binge ethanol increases brain pro-inflammatory cytokines. These results are also consistent with studies in rats showing that withdrawal from both acute and chronic ethanol increases TNFα gene expression in the brain [42, 43]. While many studies have focused on the brain pro-inflammatory response to ethanol [10, 12, 42], only a few studies have examined the brain anti-inflammatory response to ethanol [18]. These studies show that IL-4, a key anti-inflammatory cytokine, is also increased in the brain during withdrawal from acute binge ethanol. These novel findings show that alcohol increases expression of various types of cytokines in the brain. Increased anti-inflammatory cytokine expression may represent an additional level of immune dysfunction with alcohol. Indeed, dysregulated immune systems often show increases in both pro- and anti-inflammatory mediators [17]. Overall, these results show induction of both pro- and anti-inflammatory gene expression in the brain during acute binge ethanol withdrawal.

Results also showed ethanol had biphasic effects on pro-inflammatory cytokine expression in microglia-like cells in vitro, with expression decreased shortly after ethanol exposure and increased when the ethanol evaporated. However, expression of anti-inflammatory cytokines only decreased when ethanol was present and did not increase at any time point. Changes in gene expression were not due to BV2 cell death. Furthermore, increased pro-inflammatory gene expression was dependently on ethanol evaporation, as continuous ethanol exposure did not increase TNFα or Ccl2 gene expression. These results are similar to those of other studies finding biphasic effects of ethanol on human monocytes in vitro. While acute ethanol exposure decreases inflammatory signaling [44], chronic ethanol exposure enhances inflammatory signaling [19]. These data also suggest increased TNFα and Ccl2 gene expression in vivo reflect direct effects of ethanol withdrawal on microglia. Indeed, previous studies find that constant ethanol exposure can act directly on microglia to change inflammatory signaling [45]. Since ethanol did not increase BV2 anti-inflammatory gene expression in vitro, this suggests the anti-inflammatory response observed in vivo may be mediated by other cell types. Indeed, previous studies have noted that astrocytes provide an anti-inflammatory balance to the pro-inflammatory activity of microglia [46]. Overall, these data show that ethanol has biphasic effects on microglial pro-inflammatory gene expression in vitro, with no increases in anti-inflammatory gene expression.

Treating mice with the CSF1R inhibitor PLX5622 for 3 weeks led to substantial microglial depletion, consistent with other studies [28, 31]. Indeed, mRNA levels of several microglial markers were reduced by more than 90% (Fig. 6a). It is worth noting that mRNA levels of CSF1R itself were markedly reduced following PLX5622 treatment, suggesting CSF1R is located predominantly on microglia. Microglial depletion decreased TNFα expression, consistent with previous studies [28]. However, mRNA levels of IL-1β, IL-6, Ccl2, and several other immune genes were surprisingly unchanged in PLX5622-treated mice (Fig. 6a). As the main immune cells of the CNS, one might expect these genes to be primarily expressed in microglia and that their expression would decrease in the microglia-depleted brain. While immunohistochemical studies revealed small, scattered populations of microglia remaining in the brain (Fig. 5), the extent of depletion was striking. Indeed, whole brain levels of several microglial marker transcripts were reduced by more than 90% (Fig. 6a). It is possible expression of IL-1β, IL-6, Ccl2, etc. did not decrease because these genes are expressed in other brain cell types. Indeed, astrocytes have been observed to produce IL-1β [47]. Other studies suggest IL-6 is produced by many brain cell types, including neurons [48, 49], astrocytes [50], and endothelial cells [51]. Ccl2 expression has also been observed in neurons [52] and astrocytes [53]. Furthermore, the genes Ym1 and Arg1 are thought to be microglia-specific markers of the alternative or M2 activation state. However, some studies suggest astrocytes may also express Ym1 and Arg1 [54], and other studies suggest neurons express Arg1 [55]. Studies examining the transcriptome of the brain cell types also suggest that Arg1 is expressed in neurons [56]. We also examined expression of multiple TLRs. TLRs are thought to be prominently expressed in microglia [57]. PLX5622 decreased expression of TLR2 and TLR7 but surprisingly did not significantly decrease TLR3 or TLR4 expression. Indeed, microglial TLR4 is thought to play an important role in the effects of alcohol on microglia in cell culture [58]. Data from our studies in this manuscript suggest that TLR3 and TLR4 are expressed in other cell types. Indeed, TLR3 and TLR4 have been observed to co-localize with cortical neurons using immunohistochemistry [36, 59]. Furthermore, sequencing studies of the various cell types of the brain find that TLR3 and TLR4 are prominently expressed in other cell types, including astrocytes and endothelial cells [56]. Thus, our studies and others support findings of many genes previously thought to be microglial specific are likely expressed in other brain cell types as well. Overall, these results find that PLX5622 treatment decreased expression of many microglial genes and some, but not all, neuroimmune genes (Fig. 8b).

Expression of certain other genes was also impacted by microglial depletion. Neuronal markers MAP2 and DCX were unchanged, while expression of NeuN was slightly increased, consistent with other studies showing little to no effect of CSF1R inhibitors on neuronal markers [28]. PLX5622 increased the astrocyte marker GFAP and had no effect on oligodendrocyte markers MBP and CNP, consistent with previous studies [28]. PLX5622 had no effect on the endothelial cell markers Pecam1 and ICAM2. Since microglia interact extensively with the other cell types of the CNS, we also examined expression of some genes that mediate these interactions. Neurons express CD200 and CX3CL1 (also known as fractalkine). These ligands interact with their cognate receptors on microglia and function to keep the microglia in a quiescent state [37]. Interestingly, microglial depletion did not alter expression of neuronal CD200 or CX3CL1. Microglia have also been found to play an important role in synaptic pruning via components of the complement cascade [60]. Components such as C3 and C1qA “tag” neural synapses for elimination by microglia. Interestingly, PLX5622 increased expression of C3 and dramatically reduced expression of C1qA. It is possible that compensatory upregulation of C3 may be occurring due to a lack of microglial synaptic elimination. These data also suggest that C1qA is predominantly expressed in microglia. It is possible that in the absence of microglia, there may be altered synaptic pruning. Finally, since depleting microglia may change neuronal functioning, we measured the expression of four immediate-early genes, genes that increase with neuronal activation. There was no significant difference in expression of either c-Fos, EGR1, c-jun, or Arc following PLX5622 treatment. Overall, these data suggest that microglial depletion has little effect on markers of other CNS cell types but markedly alters expression of synaptic pruning genes.

These studies also examined the role of microglia in the brain response to acute binge ethanol withdrawal. These studies are, to our knowledge, the first to do so by depleting microglia in vivo. Multiple cell types of the CNS express immune genes [61], leaving it unclear which ethanol responses are due to microglia. As expected, microglial depletion reduced expression of multiple microglial genes, including Iba1, CD68, CD11b, etc. Furthermore, ethanol-induced TNFα was completely blocked by microglial depletion, suggesting ethanol-induced TNFα is microglia-derived. These results are similar to those of other studies finding microglial depletion completely blocked LPS-induced TNFα in the brain [28]. Ccl2 mRNA was partially, but not completely blocked by microglial depletion, suggesting multiple CNS cell types contribute to ethanol-induced Ccl2 expression. Surprisingly, microglial depletion did not reduce ethanol-induced IL-1β or IL-6. This finding is unexpected, as one might expect microglia to be the primary sources of these inflammatory mediators, especially following an insult. Indeed, microglial depletion blunted LPS-induced IL-1β in the brain [28]. However, microglial depletion did not decrease LPS-induced IL-6, suggesting that other CNS cell types are the primary sources of IL-6. Microglial depletion also enhanced withdrawal-induced anti-inflammatory gene expression, including IL-1ra, IL-4, IL-10, and Arg1. Since small, scattered populations of microglia remain following depletion, it is possible the remaining microglia contribute to this immune response. However, it is also possible these genes are expressed by other brain cell types, such as astrocytes, during ethanol withdrawal, and microglia normally suppress expression of these genes. These results suggest that microglia play a role mediating the balance of pro- and anti-inflammatory forces during ethanol withdrawal. Overall, these results find that microglia play a role in the neuroimmune effects of ethanol on the brain (Fig. 8c).

This study also examined the effects of microglial depletion on death receptor gene expression following acute binge ethanol. Interestingly, microglial depletion enhanced the ethanol response of multiple death receptor/ligand genes. Previous studies find that ethanol causes FasR-FasL induced cell death in the hepatocytes [62]. Furthermore, ethanol was found to increase FasL mRNA and cell death in cortical slices in vitro [63]. Since death receptors/ligands play important roles in apoptosis, these results indicate that microglial depletion may increase cell death following acute binge ethanol. While microglia are often thought to contribute to neurodegeneration [27], there is literature supporting a role for microglia in neuroprotection [26]. Indeed, it is likely that microglia can be either neurotoxic or neuroprotective depending on the circumstances. Future studies could further examine the role of microglia in ethanol-induced cell death. Overall, these results find that microglial depletion enhances the ethanol response of death receptor/ligand expression.

Our study also examined the role of microglia in the behavioral effects of acute ethanol. This was tested in two different ways: by using PLX5622 to deplete microglia and by using the recombinant IL-1ra compound, Kineret, which is believed to impact microglial function by modulating signaling of the cytokine IL-1β. We specifically used Kineret in an attempt to replicate previous studies examining the role of microglia in ethanol-induced motor impairment [30]. We investigated the impact of microglia on ethanol-induced behavioral changes using the rotarod. Microglial depletion via 1 week of PLX5622 treatment did not change ethanol-induced motor impairment at any time point (Additional file 9: Figure S9). Also, treatment with two different doses of Kineret (100 and 300 mg/kg) did not change ethanol-induced motor impairment at any time point (Additional file 9: Figure S9). These results did not replicate previous findings that Kineret increased time spent on the rotarod following ethanol [30]. This may be due to the fact that different strains of mice were used. While our study used C57BL/6 mice, the other study used Balb/c mice [30]. Overall, our results do not support the hypothesis that microglia mediate the effects of acute ethanol on motor impairment.

Furthermore, results of this study support acute ethanol withdrawal inducing a pro-inflammatory microglial phenotype. This is supported by the fact that microglia-like cells treated with ethanol in vitro show a pro-inflammatory response, but not an anti-inflammatory response, and that depleting microglia in vivo blunts the ethanol-induced pro-inflammatory response. Microglial activation phenotypes are usually described as occurring along an M1-M2 spectrum, with M1 representing the pro-inflammatory, destructive phenotype, and M2 representing the anti-inflammatory, reparative phenotype. Previous studies have found varying results regarding the microglial phenotype induced by ethanol, with some studies suggesting an M1 phenotype [40] and others suggesting an M2 phenotype [18]. Microglia have been shown to exhibit complex, dynamic responses to stimuli [41]. Therefore, it is likely that the microglial response to ethanol depends on the species being studied, as well as the dose, duration, and pattern of alcohol administration. Indeed, previous studies suggest a single ethanol binge causes a milder, homeostatic microglial activation phenotype, while a second binge causes a more robust, pro-inflammatory phenotype [64]. A complete understanding of the effects of ethanol on microglia may require examining entire microglial transcriptomes. Indeed, recent studies have called into question the M1-M2 activation schema, describing instead a complex constellation of several microglial activation states [15, 65]. Our results also question various aspects of the M1-M2 scheme, as various M1-M2 markers, such as iNOS and Arg1, were not reduced in microglia-depleted animals. Further studies will be necessary to define thoroughly the complex effects of ethanol on microglial function. Such studies will be critical, as microglia have been found to alter neuronal functioning and even behavior [20, 22], and may contribute to the development or consequences of alcoholism. Overall, these studies suggest acute ethanol withdrawal induces pro-inflammatory gene expression in microglia.

## Conclusions

We report in these studies that acute binge ethanol causes biphasic changes in microglial genes, with an initial decrease during intoxication, followed by a later increase during withdrawal. Also, acute binge ethanol withdrawal dose dependently increased neuroimmune gene expression beginning at high doses. Cultured BV2 microglia-like cells showed biphasic changes in pro-inflammatory gene expression following ethanol exposure and evaporation in vitro. Administration of PLX5622 depleted microglia from the brains of mice. Although some neuroimmune genes were reduced by microglial depletion, many others were unchanged, suggesting that the neuroimmune system involves many brain cell types. Finally, microglial depletion blunted the pro-inflammatory response and enhanced the anti-inflammatory response to acute binge ethanol withdrawal. These findings suggest microglia contribute to the impact of heavy binge ethanol on the brain, particularly during withdrawal.

## Additional files

Additional file 1: Figure S1. Schematic of experimental designs. a) For the Dose-Response Experiment, mice were gavaged with 3.0, 4.5 or 6.0 g/kg ethanol (25% v/v) and pain response and movement were qualitatively assessed each following hour. For each group, BACs were collected 1 hour post-gavage. Mice in these groups were sacrificed at 18 hours and brain mRNA was collected for RT-PCR. Note that mice in the group receiving 3.0 g/kg ethanol were not qualitatively assessed, as they exhibited only minor behavioral changes. b) For the Time Course Experiment, mice were gavaged with 6.0 g/kg ethanol (25% v/v) and sacrificed at 6, 12, 18 or 24 hours. A non-gavaged “0 hour” control group was also included. Brain mRNA was collected for RT-PCR. c) A separate group of mice was gavaged with 6.0 g/kg ethanol (25% v/v) and tail blood was collected at 1, 6, 12 and 18 hours for assessment of BACs. d) Mice were given either Control chow or PLX5622 chow. One week after starting PLX5622 chow to deplete microglia, mice were injected intraperitoneally with 2.0 g/kg ethanol (20% v/v) and tested on the rotarod. After two more weeks of treatment with PLX5622 chow, mice were gavaged with 6.0 g/kg ethanol (25% v/v) and brain mRNA was collected 18 hours later for RT-PCR. (TIF 108 kb)

Additional file 2: Figure S2. Behavioral characterization of the acute binge ethanol model. Mice were gavaged with ethanol (4.5 or 6 g/kg, 25% *v*/*v*) and pain response and movement were qualitatively assessed each following hour. Note that there was 0% mortality, as indicated by behavior recorded for 100% of mice in each group at each time point. A, C) For movement, a complete absence of movement other than breathing was recorded as “No Activity.” Head movement, limb movement, or impaired ambulation was recorded as “Impaired Activity.” Movement that was indistinguishable from a control mouse was recorded as “Full Activity.” B, D) For pain assessment, each hindpaw was pinched. A complete absence of a response was recorded as “No response.” Slight flinching or movement following any pinch was recorded as “Weak Response.” Full-paw withdrawal following any pinch was recorded as “Full Response.” *n* = 8/group (TIF 175 kb)

Additional file 3: Figure S3. Acute binge ethanol causes biphasic changes in brain Iba1 and CD68 mRNA compared to controls. Mice were gavaged with acute binge ethanol (6 g/kg, 25% *v*/*v*) or water and sacrificed various times post-treatment. A) Brain Iba1 was measured over time by RT-PCR in both water- and ethanol-treated mice. B) Brain CD68 was measured over time by RT-PCR in both water- and ethanol-treated mice. Note there is no change in brain Iba1 or CD68 mRNA in water-treated mice over time. Data are represented as mean ± SEM. *p < 0.05 compared to controls. *n* = 5–6/group (TIF 1013 kb)

Additional file 4: Figure S4. Acute binge ethanol withdrawal increases brain TNFα, Ccl2, and IL-4 mRNA compared to controls. Mice were gavaged with acute binge ethanol (6 g/kg, 25% *v*/*v*) or water and sacrificed various times post-treatment. A) Brain TNFα, B) Ccl2, C) IL-1ra, and D) IL-4 were measured over time by RT-PCR in both water- and ethanol-treated mice. Data are represented as mean ± SEM. *p < 0.05 compared to controls. *n* = 5–7/group (TIF 136 kb)

Additional file 5: Figure S5. Acute binge ethanol increases brain TNFα, Ccl2, and IL-4 protein during withdrawal. Mice were treated with ethanol (6 g/kg, 25% *v*/*v*) or water and sacrificed 18 h post-treatment. Brain protein was collected and A) TNFα, B) Ccl2, C) IL-1ra, and D) IL-4 protein levels were determined via ELISA. Data are represented as mean ± SEM. *p < 0.05, Student’s *t* test, *n* = 6/group (TIF 97 kb)

Additional file 6: Figure S6. Acute ethanol treatment and evaporation changes BV2 TNFα, Ccl2, IL-1ra, and IL-4 mRNA compared to controls. Time course of cytokine expression in ethanol-treated and PBS-treated control BV2 cells: Microglia-like BV2 cells were treated with either ethanol (85 mM) or PBS and the ethanol was allowed to evaporate away over time. Transcript levels of A) TNFα B) Ccl2 C) IL-1ra, and D) IL-4 were assessed in ethanol-treated cells (black dots) and PBS-treated control cells (white dots). Baseline changes in gene expression likely occur because the BV2 cells are proliferating and engaging in autocrine-paracrine signaling in a closed system. Data are represented as mean ± SEM. *p < 0.05, Student’s *t* test, *n* = 4–6/group (TIF 144 kb)

Additional file 7: Figure S7. BV2 viability following ethanol treatment. Microglia-like BV2 cells were treated with PBS or ethanol (85 mM) and the ethanol was allowed to evaporate away over time. At 0, 1.5, 12, and 24 h, cell viability was determined with the vital stain, Trypan blue. The number of live and dead cells was counted, and the number of live cells was divided by the number of total cells to calculate percent viability. Note that ethanol treatment did not affect cell viability at any time point. Data are represented as mean ± SEM. *n* = 3/group (TIF 514 kb)

Additional file 8: Figure S8. Continuous ethanol treatment does not increase BV2 TNFα or Ccl2 expression. Microglia-like BV2 cells were treated with either ethanol (85 mM) or PBS. A) For ethanol-treated cells, ethanol was vaporized into the incubator to keep media ethanol concentrations constant. After 24 h of continuous ethanol exposure, BV2 mRNA was isolated and B) TNFα and C) Ccl2 gene expression were measured. Note that continuous ethanol exposure does not increase BV2 pro-inflammatory cytokine expression at 24 h. Data are represented as mean ± SEM. *p < 0.05, Student’s *t* test, *n* = 6/group (TIF 94 kb)

Additional file 9: Figure S9. The role of microglia in ethanol-induced motor impairment. Mice were treated with compounds that impact microglia—either the CSF1R inhibitor PLX5622 or recombinant IL-1ra (Kineret). A) Mice were fed PLX5622 chow for 1 week to deplete microglia and injected i.p. with ethanol (2.0 g/kg, 20% *v*/*v*). Mice were then tested on the rotarod at 2, 5, 8, 14, 20 min and every subsequent 10 min post-injection until 110 min had passed. The time the mice remained on the rotarod was recorded. Note that microglial depletion with 1 week on PLX5622 chow did not alter the ethanol-induced motor impairments. B, C) Mice received an intraperitoneal injection of B) 100 or C) 300 mg/kg IL-1ra 30 min prior to an i.p. injection of ethanol (2.0 g/kg, 20% *v*/*v*). Mice were then tested on the rotarod at 2, 5, 8, 14, 20 min and every subsequent 10 min after ethanol injection until 110 min had passed. The time the mice remained on the rotarod was recorded. Note that IL-1ra did not alter the ethanol-induced motor impairments. *n* = 8/group (TIF 147 kb)

Additional file 10: Table S1. Effects of acute binge ethanol on brain gene expression over time (DOC 53 kb)

Additional file 11: Table S2. Effects of acute binge ethanol dose on brain gene expression during withdrawal (DOC 36 kb)

Additional file 12: Table S3. Effects of ethanol treatment on BV2 gene expression over time (DOC 47 kb)

## Abbreviations

AUD: Alcohol use disorder
BAC: Blood alcohol concentration
CSF1R: Colony stimulating factor 1 receptor
MEC: Media ethanol concentration
