# Supplementary figures and images for: Microglial depletion alters the brain neuroimmune response to acute binge ethanol withdrawal
Source: J Neuroinflammation. 2017 Apr 20;14:86. doi: 10.1186/s12974-017-0856-z (PMC5439231; doi:10.1186/s12974-017-0856-z)

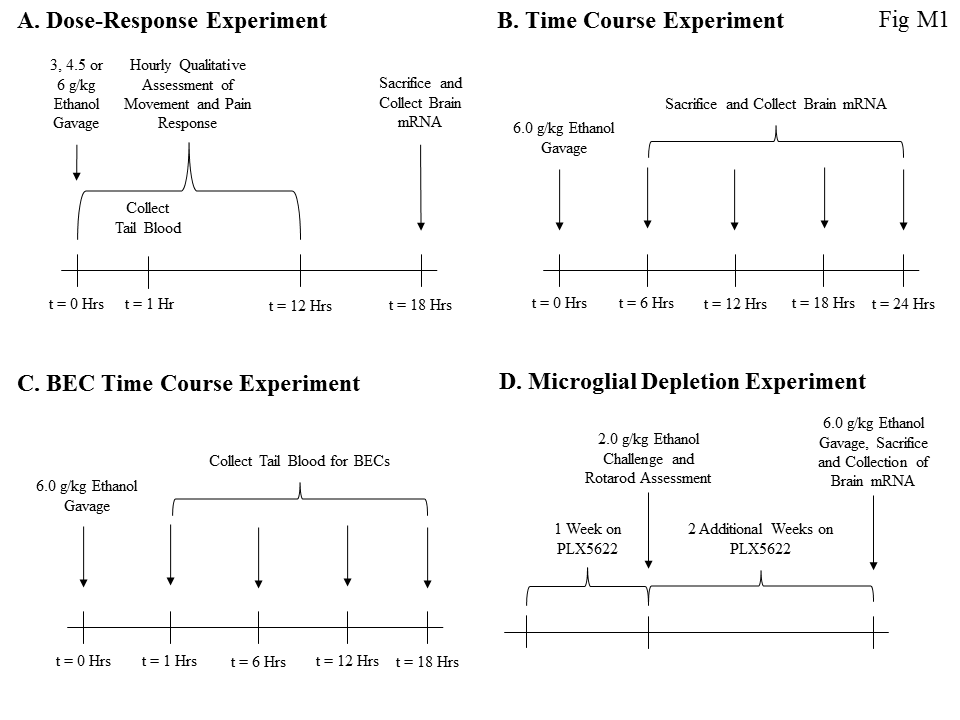

Supplement: Supplementary file 1 — Schematic of experimental designs. a) For the Dose-Response Experiment, mice were gavaged with 3.0, 4.5 or 6.0 g/kg ethanol (25% v/v) and pain response and movement were qualitatively assessed each following hour. For each group, BACs were collected 1 hour post-gavage. Mice in these groups were sacrificed at 18 hours and brain mRNA was collected for RT-PCR. Note that mice in the group receiving 3.0 g/kg ethanol were not qualitatively assessed, as they exhibited only minor behavioral changes. b) For the Time Course Experiment, mice were gavaged with 6.0 g/kg ethanol (25% v/v) and sacrificed at 6, 12, 18 or 24 hours. A non-gavaged “0 hour” control group was also included. Brain mRNA was collected for RT-PCR. c) A separate group of mice was gavaged with 6.0 g/kg ethanol (25% v/v) and tail blood was collected at 1, 6, 12 and 18 hours for assessment of BACs. d) Mice were given either Control chow or PLX5622 chow. One week after starting PLX5622 chow to deplete microglia, mice were injected intraperitoneally with 2.0 g/kg ethanol (20% v/v) and tested on the rotarod. After two more weeks of treatment with PLX5622 chow, mice were gavaged with 6.0 g/kg ethanol (25% v/v) and brain mRNA was collected 18 hours later for RT-PCR. (TIF 108 kb) [file 12974_2017_856_MOESM1_ESM.tif]

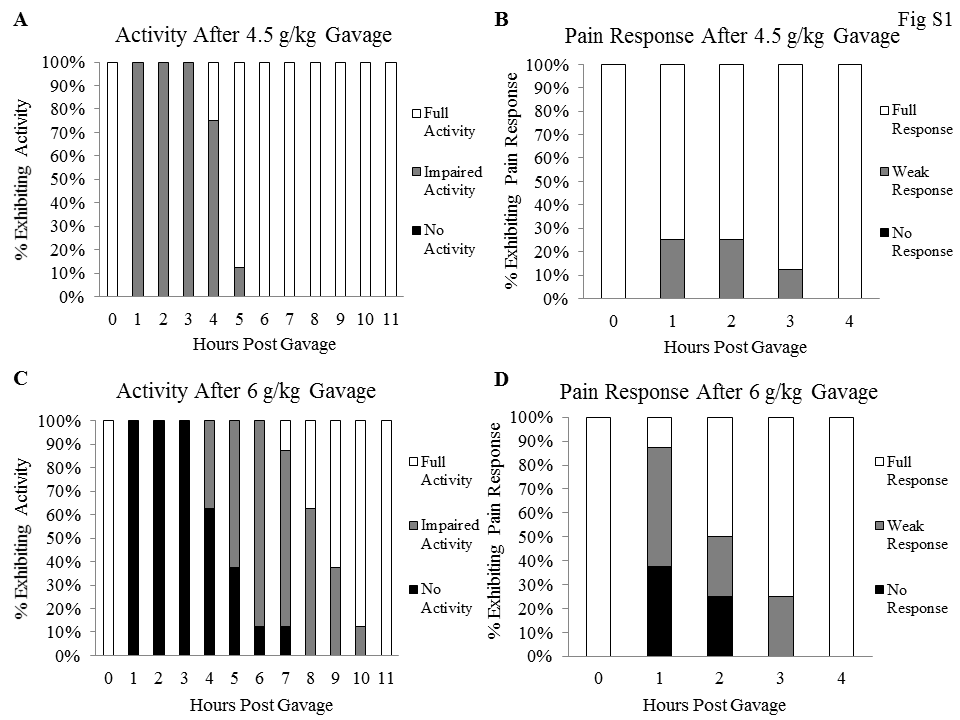

Supplement: Supplementary file 2 — Behavioral characterization of the acute binge ethanol model. Mice were gavaged with ethanol (4.5 or 6 g/kg, 25% v/v) and pain response and movement were qualitatively assessed each following hour. Note that there was 0% mortality, as indicated by behavior recorded for 100% of mice in each group at each time point. A, C) For movement, a complete absence of movement other than breathing was recorded as “No Activity.” Head movement, limb movement, or impaired ambulation was recorded as “Impaired Activity.” Movement that was indistinguishable from a control mouse was recorded as “Full Activity.” B, D) For pain assessment, each hindpaw was pinched. A complete absence of a response was recorded as “No response.” Slight flinching or movement following any pinch was recorded as “Weak Response.” Full-paw withdrawal following any pinch was recorded as “Full Response.” n = 8/group (TIF 175 kb) [file 12974_2017_856_MOESM2_ESM.tif]

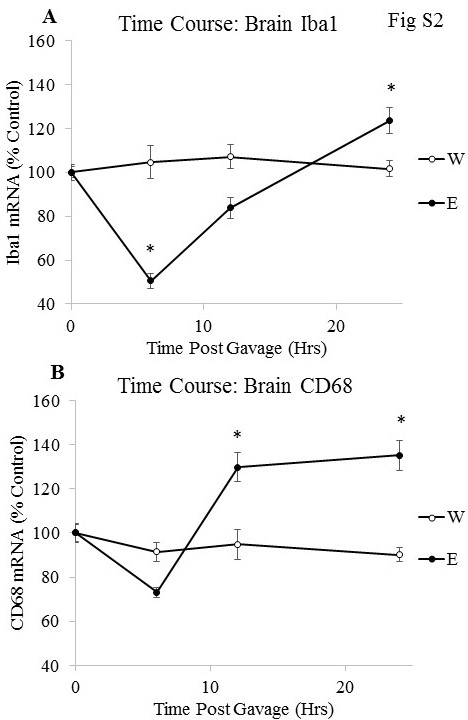

Supplement: Supplementary file 3 — Acute binge ethanol causes biphasic changes in brain Iba1 and CD68 mRNA compared to controls. Mice were gavaged with acute binge ethanol (6 g/kg, 25% v/v) or water and sacrificed various times post-treatment. A) Brain Iba1 was measured over time by RT-PCR in both water- and ethanol-treated mice. B) Brain CD68 was measured over time by RT-PCR in both water- and ethanol-treated mice. Note there is no change in brain Iba1 or CD68 mRNA in water-treated mice over time. Data are represented as mean ± SEM. *p < 0.05 compared to controls. n = 5–6/group (TIF 1013 kb) [file 12974_2017_856_MOESM3_ESM.tif]

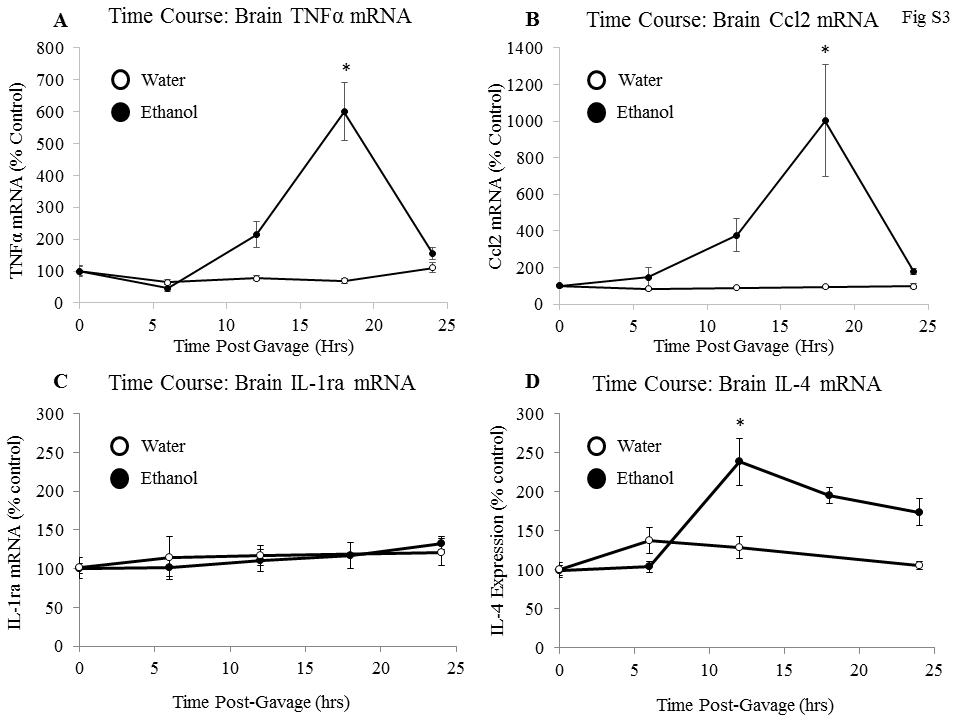

Supplement: Supplementary file 4 — Acute binge ethanol withdrawal increases brain TNFα, Ccl2, and IL-4 mRNA compared to controls. Mice were gavaged with acute binge ethanol (6 g/kg, 25% v/v) or water and sacrificed various times post-treatment. A) Brain TNFα, B) Ccl2, C) IL-1ra, and D) IL-4 were measured over time by RT-PCR in both water- and ethanol-treated mice. Data are represented as mean ± SEM. *p < 0.05 compared to controls. n = 5–7/group (TIF 136 kb) [file 12974_2017_856_MOESM4_ESM.tif]

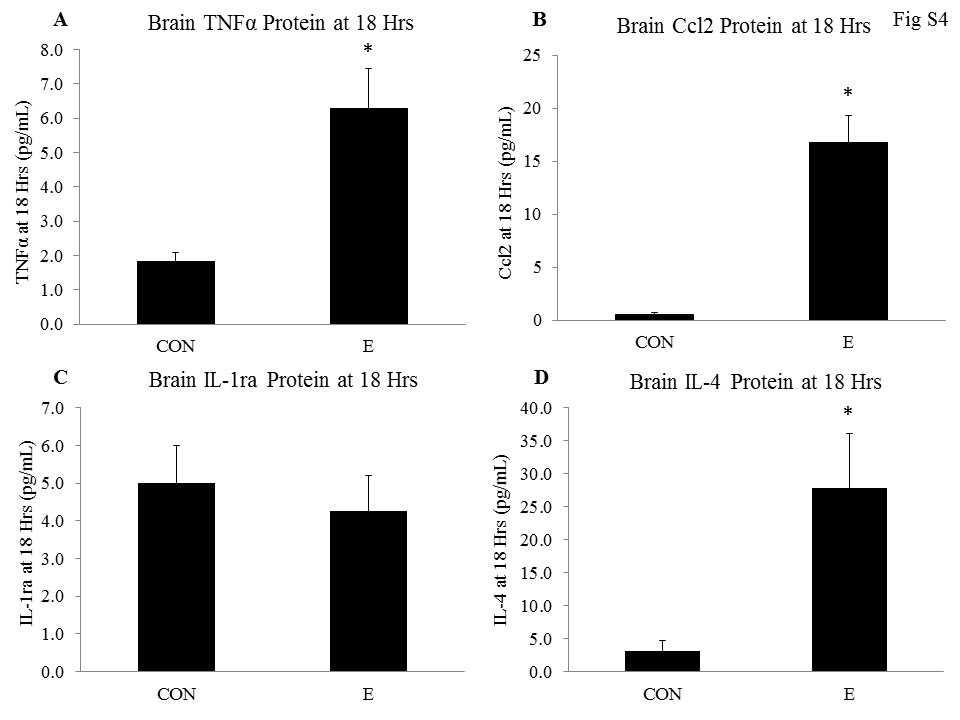

Supplement: Supplementary file 5 — Acute binge ethanol increases brain TNFα, Ccl2, and IL-4 protein during withdrawal. Mice were treated with ethanol (6 g/kg, 25% v/v) or water and sacrificed 18 h post-treatment. Brain protein was collected and A) TNFα, B) Ccl2, C) IL-1ra, and D) IL-4 protein levels were determined via ELISA. Data are represented as mean ± SEM. *p < 0.05, Student’s t test, n = 6/group (TIF 97 kb) [file 12974_2017_856_MOESM5_ESM.tif]

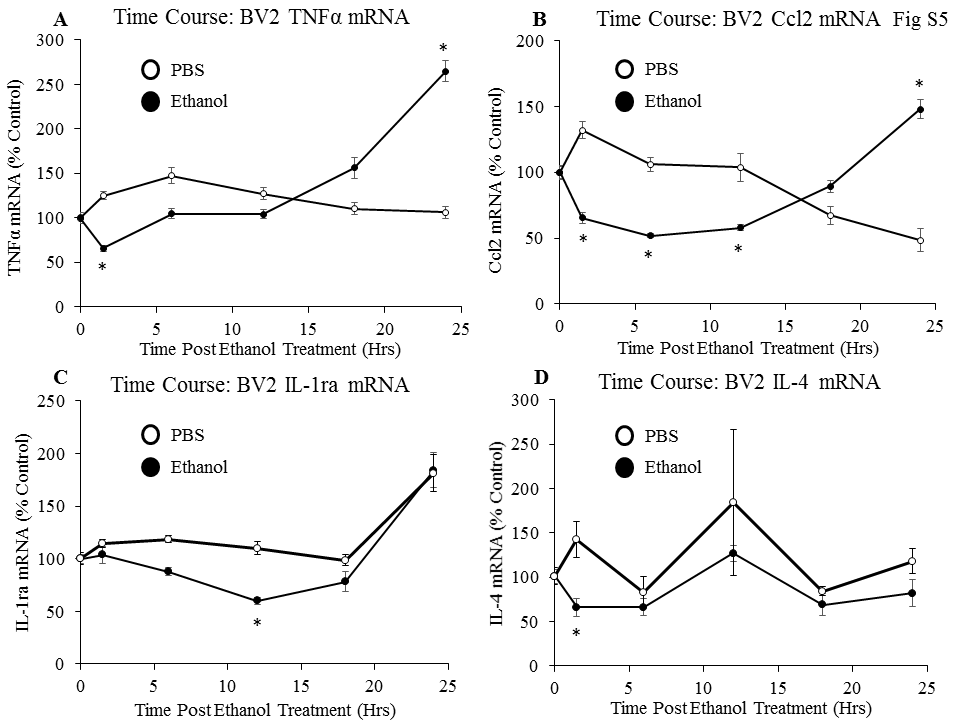

Supplement: Supplementary file 6 — Acute ethanol treatment and evaporation changes BV2 TNFα, Ccl2, IL-1ra, and IL-4 mRNA compared to controls. Time course of cytokine expression in ethanol-treated and PBS-treated control BV2 cells: Microglia-like BV2 cells were treated with either ethanol (85 mM) or PBS and the ethanol was allowed to evaporate away over time. Transcript levels of A) TNFα B) Ccl2 C) IL-1ra, and D) IL-4 were assessed in ethanol-treated cells (black dots) and PBS-treated control cells (white dots). Baseline changes in gene expression likely occur because the BV2 cells are proliferating and engaging in autocrine-paracrine signaling in a closed system. Data are represented as mean ± SEM. *p < 0.05, Student’s t test, n = 4–6/group (TIF 144 kb) [file 12974_2017_856_MOESM6_ESM.tif]

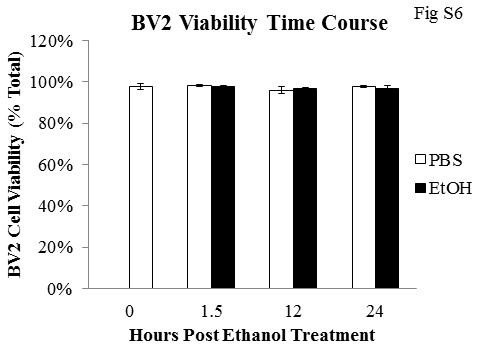

Supplement: Supplementary file 7 — BV2 viability following ethanol treatment. Microglia-like BV2 cells were treated with PBS or ethanol (85 mM) and the ethanol was allowed to evaporate away over time. At 0, 1.5, 12, and 24 h, cell viability was determined with the vital stain, Trypan blue. The number of live and dead cells was counted, and the number of live cells was divided by the number of total cells to calculate percent viability. Note that ethanol treatment did not affect cell viability at any time point. Data are represented as mean ± SEM. n = 3/group (TIF 514 kb) [file 12974_2017_856_MOESM7_ESM.tif]

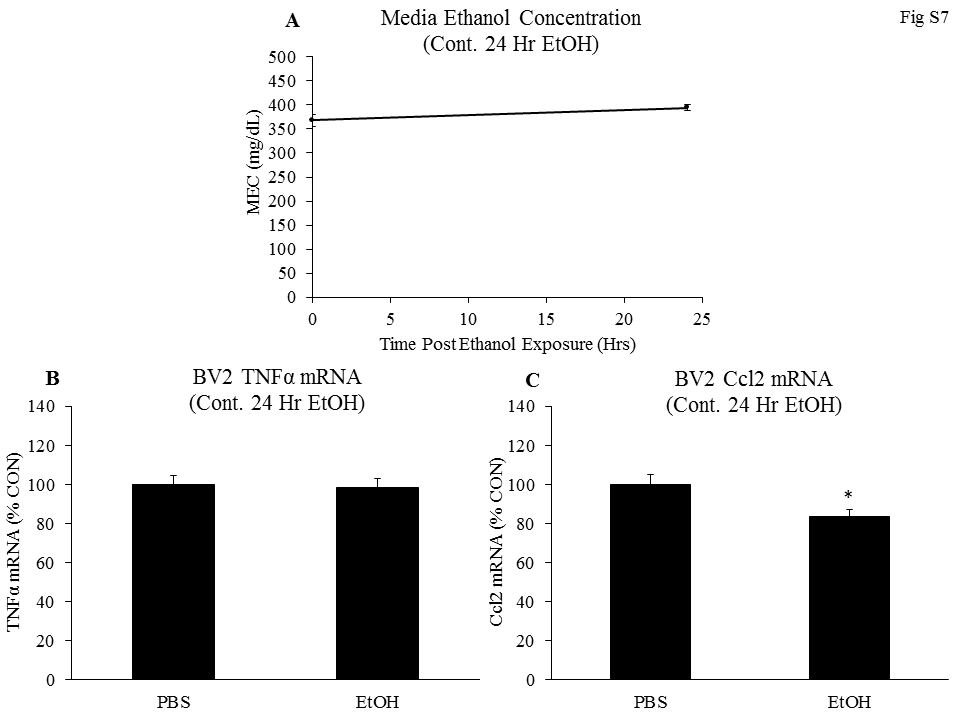

Supplement: Supplementary file 8 — Continuous ethanol treatment does not increase BV2 TNFα or Ccl2 expression. Microglia-like BV2 cells were treated with either ethanol (85 mM) or PBS. A) For ethanol-treated cells, ethanol was vaporized into the incubator to keep media ethanol concentrations constant. After 24 h of continuous ethanol exposure, BV2 mRNA was isolated and B) TNFα and C) Ccl2 gene expression were measured. Note that continuous ethanol exposure does not increase BV2 pro-inflammatory cytokine expression at 24 h. Data are represented as mean ± SEM. *p < 0.05, Student’s t test, n = 6/group (TIF 94 kb) [file 12974_2017_856_MOESM8_ESM.tif]

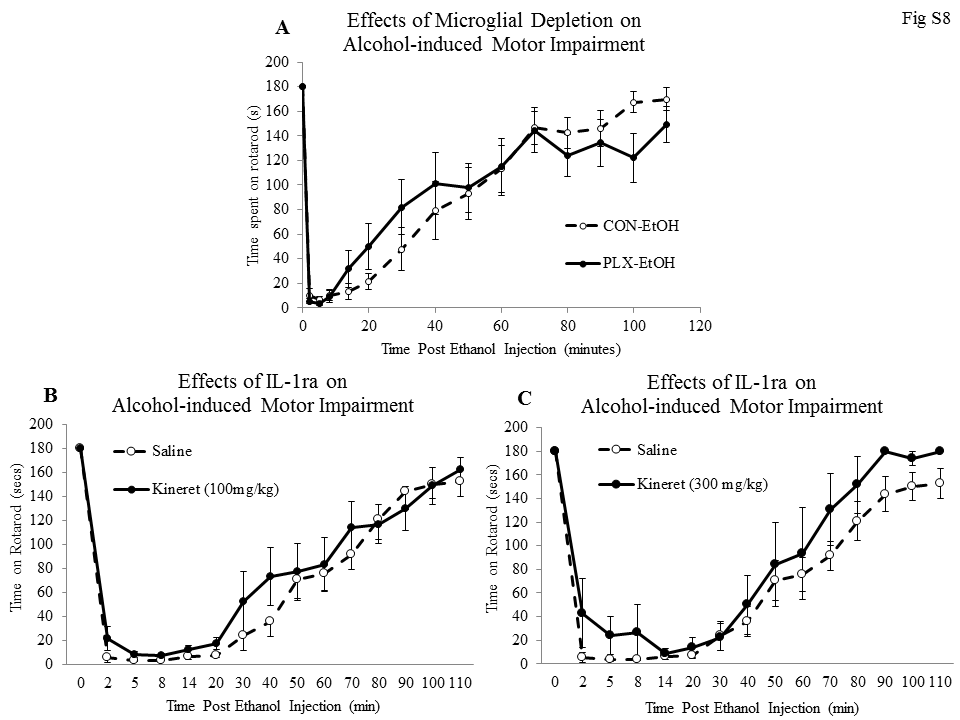

Supplement: Supplementary file 9 — The role of microglia in ethanol-induced motor impairment. Mice were treated with compounds that impact microglia—either the CSF1R inhibitor PLX5622 or recombinant IL-1ra (Kineret). A) Mice were fed PLX5622 chow for 1 week to deplete microglia and injected i.p. with ethanol (2.0 g/kg, 20% v/v). Mice were then tested on the rotarod at 2, 5, 8, 14, 20 min and every subsequent 10 min post-injection until 110 min had passed. The time the mice remained on the rotarod was recorded. Note that microglial depletion with 1 week on PLX5622 chow did not alter the ethanol-induced motor impairments. B, C) Mice received an intraperitoneal injection of B) 100 or C) 300 mg/kg IL-1ra 30 min prior to an i.p. injection of ethanol (2.0 g/kg, 20% v/v). Mice were then tested on the rotarod at 2, 5, 8, 14, 20 min and every subsequent 10 min after ethanol injection until 110 min had passed. The time the mice remained on the rotarod was recorded. Note that IL-1ra did not alter the ethanol-induced motor impairments. n = 8/group (TIF 147 kb) [file 12974_2017_856_MOESM9_ESM.tif]
