# Supplementary material for: Microglial depletion alters the brain neuroimmune response to acute binge ethanol withdrawal
Source: J Neuroinflammation. 2017 Apr 20;14:86. doi: 10.1186/s12974-017-0856-z (PMC5439231; doi:10.1186/s12974-017-0856-z)
Supplement: Supplementary file 10 — Effects of acute binge ethanol on brain gene expression over time (DOC 53 kb) [file 12974_2017_856_MOESM10_ESM.doc]

**Table S1. Effects of Acute Binge Ethanol on Brain Gene Expression over Time.**

|  | **0 Hr** | **6 Hrs** | **12 Hrs** | **24 Hrs** | **48 Hrs** |
| --- | --- | --- | --- | --- | --- |
| **Microglial Markers** | | | | |  |
| Iba1 | 100 ± 3 | 51 ± 4* | 84 ± 5 | 123 ± 6* | 103 ± 4 |
| CD11b | 100 ± 8 | 74 ± 5* | 65 ± 3* | 84 ± 7 | 102 ± 4 |
| **M1 Microglial Markers** | | | | |  |
| CD68 | 100 ± 4 | 73 ± 2* | 130 ± 7* | 135 ± 7* | 111 ± 4 |
| CD86 | 100 ± 5 | 115 ± 5 | 129 ± 4* | 118 ± 7 | 107 ± 8 |
| iNOS | 100 ± 6 | 31 ± 5* | 92 ± 7 | 106 ± 6 | 90 ± 7 |
| NOX2 | 100 ± 24 | 113 ± 18 | 148 ± 31 | 135 ± 23 | 116 ± 31 |
| **M2 Microglial Markers** | | | | |  |
| CD206 | 100 ± 8 | 88 ± 3 | 94 ± 8 | 106 ± 6 | 84 ± 4 |
| CD163 | 100 ± 7 | 224 ± 9* | 116 ± 13 | 104 ± 7 | 118 ± 9 |
| Arg1 | 100 ± 7 | 95 ± 9 | 95 ± 16 | 98 ± 7 | 86 ± 10 |
| Ym1 | 100 ± 8 | 162 ± 25 | 212 ± 47 | 195 ± 44 | 116 ± 12 |
| **Pro-inflammatory Cytokines** | | | | |  |
| IL-1β | 100 ± 3 | 94 ± 7 | 112 ± 12 | 117 ± 6 | 95 ± 7 |
| TNFα | 100 ± 17 | 46 ± 10 | 214 ± 41* | 154 ± 18 | 119 ± 14 |
| IL-6 | 100 ± 6 | 83 ± 8 | 105 ± 10 | 125 ± 7 | 109 ± 13 |
| Ccl2 | 100 ± 9 | 148 ± 54 | 376 ± 89* | 178 ± 18 | 147 ± 24 |
| **Anti-inflammatory Cytokines** | | | | |  |
| IL-10 | 100 ± 5 | 99 ± 9 | 106 ± 13 | 126 ± 3 | 108 ± 14 |
| IL-4 | 100 ± 6 | 104 ± 7 | 238 ± 30* | 174 ± 17* | 105 ± 8 |
| IL-1ra | 100 ± 6 | 102 ± 12 | 111 ± 14 | 132 ± 8 | 112 ± 13 |
| TGF-β1 | 100 ± 7 | 68 ± 2* | 121 ± 14 | 96 ± 5 | 95 ± 6 |
| **CNS Cell Types** | | | | |  |
| NeuN | 100 ± 7 | 104 ± 7 | 108 ± 4 | 100 ± 4 | 117 ± 2 |
| MAP2 | 100 ± 3 | 84 ± 2* | 106 ± 6 | 95 ± 3 | 97 ± 3 |
| GFAP | 100 ± 10 | 81 ± 7 | 128 ± 19 | 125 ± 12 | 102 ± 7 |
| MBP | 100 ± 10 | 101 ± 7 | 104 ± 6 | 99 ± 4 | 100 ± 5 |
| **Inflammation** | | | | |  |
| HMGB1 | 100 ± 9 | 96 ± 9 | 89 ± 5 | 118 ± 4 | 109 ± 7 |
| **Neuron-Microglia Signaling** | | | | |  |
| CX3CL1 | 100 ± 4 | 91 ± 4 | 108 ± 5 | 118 ± 8 | 84 ± 5 |
| CX3CR1 | 100 ± 3 | 43 ± 5* | 86 ± 6 | 97 ± 5 | 92 ± 4 |
| CD200 | 100 ± 6 | 107 ± 5 | 94 ± 3 | 99 ± 4 | 90 ± 3 |
| CD200R1 | 100 ± 18 | 90 ± 7 | 80 ± 7 | 94 ± 12 | 91 ± 10 |
| **Neurotrophins** | | | | |  |
| BDNF | 100 ± 7 | 66 ± 2* | 124 ± 8 | 129 ± 8* | 89 ± 4 |
| NGF | 100 ± 3 | 90 ± 2 | 115 ± 8 | 111 ± 4 | 102 ± 4 |
| NT3 | 100 ± 11 | 114 ± 10 | 93 ± 16 | 88 ± 7 | 86 ± 8 |
| NT4/5 | 100 ± 10 | 77 ± 3 | 103 ± 6 | 119 ± 9 | 115 ± 10 |
| **Neurogenesis** | | | | |  |
| DCX | 100 ± 5 | 85 ± 2 | 106 ± 8 | 97 ± 10 | 102 ± 4 |

Mice were gavaged with acute binge ethanol (6 g/kg, 25% v/v) and sacrificed 6, 12, 24 or 48 hours post-treatment. A non-gavaged “0” hour group was included as a control. * = p<0.05, ANOVA followed by Dunnett’s post-hoc test compared to 0 hr control.
