# Supplementary material for: Microglial depletion alters the brain neuroimmune response to acute binge ethanol withdrawal
Source: J Neuroinflammation. 2017 Apr 20;14:86. doi: 10.1186/s12974-017-0856-z (PMC5439231; doi:10.1186/s12974-017-0856-z)
Supplement: Supplementary file 11 — Effects of acute binge ethanol dose on brain gene expression during withdrawal (DOC 36 kb) [file 12974_2017_856_MOESM11_ESM.doc]

**Table S2. Effects of Acute Binge Ethanol Dose on Brain Gene Expression During Withdrawal.**

|  | **CON** | **3 g/kg** | **4.5 g/kg** | **6 g/kg** |
| --- | --- | --- | --- | --- |
| **M1 Microglial Markers** | | | | |
| CD68 | 100 ± 5 | 85 ± 2 | 127 ± 7* | 133 ± 5* |
| CD86 | 100 ± 8 | 118 ± 4 | 116 ± 7 | 127 ± 9 |
| **M2 Microglial Markers** | | | | |
| CD206 | 100 ± 7 | 102 ± 8 | 100 ± 6 | 84 ± 6 |
| CD163 | 100 ± 11 | 85 ± 4 | 97 ± 4 | 118 ± 9 |
| **Pro-inflammatory Cytokines** | | | | |
| IL-1β | 100 ± 10 | 100 ± 10 | 151 ± 10* | 130 ± 13 |
| TNFα | 100 ± 8 | 118 ± 8 | 218 ± 33 | 672 ± 93* |
| IL-6 | 100 ± 9 | 125 ± 15 | 186 ± 11* | 140 ± 10 |
| Ccl2 | 100 ± 8 | 84 ± 7 | 236 ± 126 | 873±290* |
| **Anti-inflammatory Cytokines** | | | | |
| IL-10 | 100 ± 10 | 101 ± 13 | 141 ± 7* | 122 ± 9 |
| IL-4 | 100 ± 14 | 136 ± 12 | 193 ± 16* | 224 ± 16* |
| IL-1ra | 100 ± 10 | 116 ± 16 | 170 ± 12* | 136 ± 14 |
| **Clock Genes** |  |  |  |  |
| Per1 | 100 ± 6 | 102 ± 4 | 185 ± 13* | 204 ± 9* |

Mice were gavaged with water or acute binge ethanol (6 g/kg, 25% v/v) and sacrificed 18 hours post-treatment. * = p<0.05, ANOVA followed by Dunnett’s *post-hoc* test compared to water gavaged control.
