# Supplementary material for: Microglial depletion alters the brain neuroimmune response to acute binge ethanol withdrawal
Source: J Neuroinflammation. 2017 Apr 20;14:86. doi: 10.1186/s12974-017-0856-z (PMC5439231; doi:10.1186/s12974-017-0856-z)
Supplement: Supplementary file 12 — Effects of ethanol treatment on BV2 gene expression over time (DOC 47 kb) [file 12974_2017_856_MOESM12_ESM.doc]

**Table S3. Effects of Ethanol Treatment on BV2 Gene Expression Over Time.**

|  | **0 Hr** | **1.5 Hrs** | **6 Hrs** | **12 Hrs** | **18 Hrs** | **24 Hrs** |
| --- | --- | --- | --- | --- | --- | --- |
| **Microglial Markers** | | | | | | |
| Iba1 | 100 ± 5 | 99 ± 3 | 91 ± 7 | 81 ± 4 | 90 ± 4 | 111 ± 9 |
| CD11b | 100 ± 4 | 94 ± 5 | 114 ± 5 | 133 ± 9* | 204 ± 2* | 285±12* |
| **M1 Microglial Markers** | | | | |  |  |
| CD68 | 100 ± 5 | 105 ± 4 | 113 ± 6 | 114 ± 6 | 130 ± 5* | 155 ± 8* |
| CD86 | 100 ± 4 | 109 ± 4 | 141 ± 5* | 156 ± 5* | 161 ± 6* | 172 ± 7* |
| iNOS | 100 ± 28 | 48 ± 4 | 59 ± 8 | 34 ± 4 | 28 ± 4 | 31 ± 7 |
| NOX2 | 100 ± 4 | 90 ± 4 | 89 ± 3 | 102 ± 5 | 137 ± 6* | 174 ± 8* |
|  |  |  |  |  |  |  |
| **M2 Microglial Markers** | | | | |  |  |
| CD206 | 100 ± 2 | 99 ± 3 | 104 ± 4 | 117 ± 6 | 144 ± 12* | 152 ± 7* |
| CD163 | 100 ± 11 | 79 ± 19 | 106 ± 18 | 83 ± 6 | 111 ± 26 | 126 ± 8 |
| Arg1 | 100 ± 10 | 109 ± 8 | 106 ± 13 | 90 ± 9 | 95 ± 9 | 105 ± 7 |
| Ym1 | 100 ± 11 | 77 ± 6 | 73 ± 6 | 90 ± 11 | 60 ± 7 | 46 ± 10* |
|  |  |  |  |  |  |  |
| **Pro-inflammatory Cytokines** | | | | |  |  |
| IL-1β | 100 ± 2 | 113 ± 4 | 92 ± 4 | 107 ± 4 | 135 ± 12 | 226 ± 20 |
| TNFα | 100 ± 6 | 66 ± 3* | 105 ± 6 | 104 ± 5 | 156 ± 12* | 265±11* |
| IL-6 | 100 ± 10 | 94 ±5 | 121 ± 10 | 123 ± 7 | 133 ± 10 | 213±27* |
| Ccl2 | 100 ± 5 | 65 ± 4* | 52 ± 2* | 58 ± 2* | 89 ± 5 | 148 ± 7* |
|  |  |  |  |  |  |  |
| **Anti-inflammatory Cytokines** | | | | |  |  |
| IL-10 | 100 ± 8 | 139 ± 9 | 155 ± 9* | 146 ± 9* | 200 ± 7* | 209±18* |
| TGF-β1 | 100 ± 6 | 97 ± 3 | 97 ± 4 | 104 ± 7 | 117 ± 11 | 121±10 |
|  | | | | |  |  |
| **Inflammation** | | | | |  |  |
| HMGB1 | 100 ± 5 | 103 ± 5 | 97 ± 4 | 70 ± 5 | 60 ± 5 | 61 ± 4 |

BV2 microglia-like cells were treated with ethanol (85 mM) *in vitro*. mRNA was collected at 0, 1.5, 6, 12, 18 and 24 hours and the expression of various genes was assessed. * = p<0.05, ANOVA followed by Dunnett’s *post-hoc* test compared to 0 hr control.
